# Increasing morphological disparity and decreasing optimality for jaw speed and strength during the radiation of jawed vertebrates

**DOI:** 10.1126/sciadv.abl3644

**Published:** 2022-03-18

**Authors:** William J. Deakin, Philip S. L. Anderson, Wendy den Boer, Thomas J. Smith, Jennifer J. Hill, Martin Rücklin, Philip C. J. Donoghue, Emily J. Rayfield

**Affiliations:** 1 School of Earth Sciences, University of Bristol, Life Sciences Building, Tyndall Avenue, Bristol BS8 1TQ, UK.; 2 Department of Evolution, Ecology and Behavior, University of Illinois, Urbana-Champaign, IL, USA.; 3 Swedish Museum of Natural History, Department of Palaeobiology, Frescativägen 40, 114 18 Stockholm, Sweden.; 4 Smithsonian Institution, National Museum of Natural History, Washington, DC 20013-7012, USA.; 5 Naturalis Biodiversity Center, Postbus 9517, 2300 RA Leiden, Netherlands.

## Abstract

The Siluro-Devonian adaptive radiation of jawed vertebrates, which underpins almost all living vertebrate biodiversity, is characterized by the evolutionary innovation of the lower jaw. Multiple lines of evidence have suggested that the jaw evolved from a rostral gill arch, but when the jaw took on a feeding function remains unclear. We quantified the variety of form in the earliest jaws in the fossil record from which we generated a theoretical morphospace that we then tested for functional optimality. By drawing comparisons with the real jaw data and reconstructed jaw morphologies from phylogenetically inferred ancestors, our results show that the earliest jaw shapes were optimized for fast closure and stress resistance, inferring a predatory feeding function. Jaw shapes became less optimal for these functions during the later radiation of jawed vertebrates. Thus, the evolution of jaw morphology has continually explored previously unoccupied morphospace and accumulated disparity through time, laying the foundation for diverse feeding strategies and the success of jawed vertebrates.

## Teaser

Jaws of the earliest jawed vertebrates were optimized for a trade-off between speed and strength.

## INTRODUCTION

**Fig. 1.**
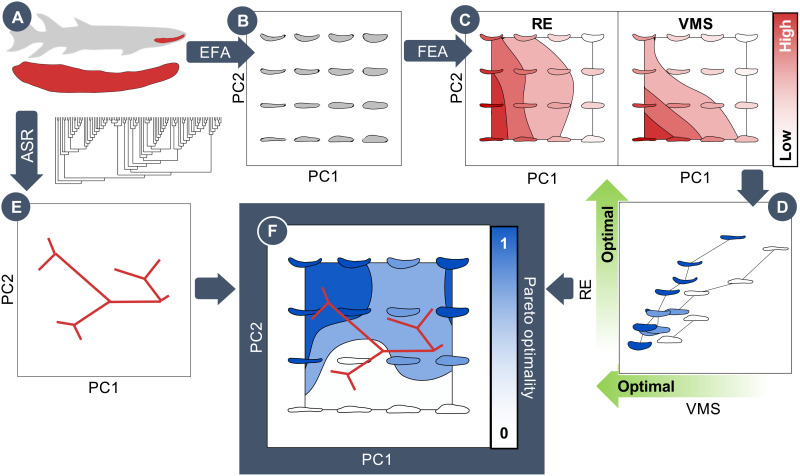
Example pipeline for adaptive landscape generation using Pareto methods. (**A**) Lateral images of 121 gnathostome jaws were collected and characterized via EFA. (**B**) EFA results were input to a PCA to build a theoretical morphospace of evenly spaced theoretical jaw shapes. (**C**) Each theoretical shape is tested 1000 times with random input constraints using finite element analysis (FEA) to assess their functional performance in RE and VMS. (**D**) Each shape was plotted in performance space with its individual performance metrics. These shapes are then ranked using a Pareto system, with the assumption that lower VMS (higher strength) is optimal and that higher RE (higher speed and efficiency) is optimal. (**E**) Ancestral state reconstruction (ASR) is used with EFA data and a timed phylogeny to construct a phylomorphospace. (**F**) The Pareto rank from the performance space is used to construct an adaptive landscape, and the evolution of taxa within this adaptive landscape is observed via the phylomorphospace.

Almost all living vertebrates are jawed vertebrates (*1*). The origin and early evolution of jaws is among the most formative of events in vertebrate evolutionary history, precipitating profound changes in predator-prey relationships and the foundations of extant vertebrate biodiversity (*1*, *2*). Many biomechanically novel feeding behaviors were established early in the evolution of jawed vertebrates, including complex linkage systems and high stress mitigation for durophagy and processing armored prey (*3*–*5*), all of which have contributed to the ecological success of vertebrates (*1*). The tempo and mode of this evolutionary episode remains poorly characterized, but those few studies that have investigated jaw functional disparity have perceived stasis throughout much of the initial gnathostome radiation (*6*, *7*), apparently contradicting the traditional view that gnathostome diversification was predicated on the innovation and broad ecological utility of the jaw. Here, we use a new approach to investigate this apparent limit on the evolution of the jaw via characterizing the range of theoretical forms accessible to early gnathostomes. We assess the functional capability of these theoretical gnathostome jaw shapes, testing which shapes are optimal for stress resistance, speed of closure, or a trade-off between these two traits. Following this, we document the temporal distribution of empirical jaw morphologies and those of inferred ancestors within theoretical morphospace to test whether gnathostome jaws were constrained by functional optimality during their early evolution. We evaluate the empirical record of jaw evolution in early jawed vertebrates within this functional context. We characterized the mandibular morphology of 121 early gnathostomes (late Silurian to the end Devonian; ~427 to 359 million years (Ma) ago through elliptical Fourier analysis (EFA) (*8*) of two-dimensional (2D) lateral images. We then generated a theoretical morphospace of mandible morphologies representing the tangent space of empirical shapes (*9*–*11*). We used functional testing to establish how strength and rotational efficiency (RE) vary through theoretical shape space, as these metrics are critical to feeding function; speed of jaw closure has been implicated in capture of fast-moving prey, while jaw strength has been linked to bite ability and the procurement of harder foodstuffs (*12*, *13*). The two traits may trade-off due to the lever-like nature of the vertebrate jaw typically considered to generate fast versus forceful closure, although similar output metrics can be reached via different morphologies [e.g., (*14*)]. This allowed us to establish an adaptive landscape within the theoretical morphospace, as has been effective in previous studies constructing performance surfaces and adaptive landscapes (*13*, *15*–*19*). However, our adaptive landscape is constructed differently to those studies that fit models to performance data. Instead, we use a new Pareto ranking approach (see Methods) that highlights optimal morphologies without using fitness functions weighted toward particular ecologies or groups (*20*, *21*), providing a general picture of optimality among extinct taxa, where survival data (*22*) are unavailable and ecological data are incomplete (see Fig. 1 for an overview of methods). Pareto concepts have been used before in the interpretation of morphospace and adaptive landscapes; however, the construction of adaptive landscapes within a Pareto framework has not been previously used (*15*–*17*, *19*, *23*).

Our metric of optimality refers to functional optimality, not true “fitness,” which is much more complex. Using a phylomorphospace approach in which we model the mandibular shapes for the ancestors of the earliest known jawed vertebrates, we characterized the phylogenetic exploration of this adaptive landscape, demonstrating that the earliest mandibles exhibited morphologies that were optimal for strength and RE. Furthermore, far from plateauing, early jawed vertebrates explored an increasing range of jaw morphospace that tracked optimal adaptive regions early in their evolution. However, subsequent sarcopterygian evolution is characterized by a shift toward less optimal regions of morphospace, perhaps reflecting a shift away from the functional drivers that characterize the initial gnathostome radiation or that functional constraints weaken over time, becoming less restrictive in the evolution of jaw form.

## RESULTS

### Theoretical morphology

**Fig. 2.**
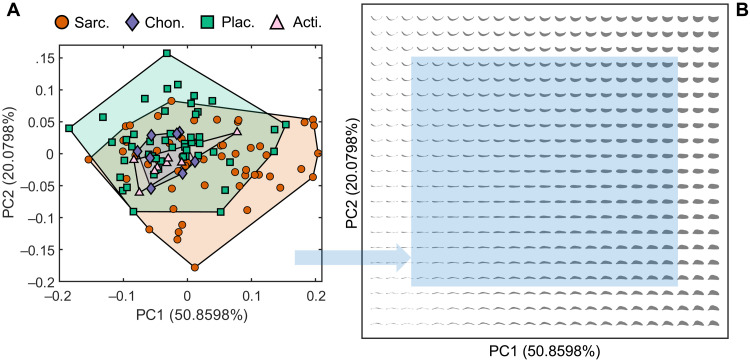
Empirical and theoretical morphospace. Theoretical morphospace (**B**) is built by extending the limits of empirical morphospace (**A**) and calculating the shape data at each point in a regular 23-by-21 grid. Legend shows symbols and colors for individual taxa from four clades: Sarcopterygii (Sarc.), Chondrichthyes and acanthodians (Chon.), Placodermi (Plac.), and Actinopterygii (Acti.). Blue area in theoretical morphospace represents the extent of empirical morphospace.

We used 12 size and rotation-corrected elliptical harmonics (45 metrics in total) output from EFA to characterize 121 gnathostome jaw shapes in 2D (Figs. 1 and 2). A principal components analysis (PCA) of the empirical shapes captures 88.6% of variation by the first five PC axes and 70.9% in the first two axes. By modifying the harmonic dataset, 483 theoretical shapes were generated in an evenly spaced 23-by-21 grid across the PC1-PC2 morphospace within an area that encompassed the range of realized jaw form plus a border of an extra 20% the range of PC1 (Fig. 2). This meets the expectations of a theoretical morphospace, since its dimensions are geometric models of form and it encompasses morphological variation that extends beyond that observed in empirical data (*10*). It is commonly argued that theoretical morphospaces are not constructed with reference to measurement data from existing form (*10*) as ours is. However, we reject this qualification, since, from their inception, theoretical morphospaces have been based on measurement data from existing form. For example, the seminal theoretical morphospace analyses by Raup (*24*, *25*) were preceded and explicitly informed by his characterization of the coiling parameters of gastropods based on empirical measurement data (*26*). Our approach allows for a much broader application of the theoretical morphospace approach, which has largely been limited to geometrically simple biological structures [e.g., (*10*)].

Within this article, we refer to jaw shapes by their position in theoretical morphospace, which spans from −0.26 to 0.28 in PC1 and −0.26 to 0.24 in PC2. In general, increasing PC1 represents increasing jaw depth and decreasing jaw length, while increasing PC2 represents the shift from a more convex to a more concave dorsal surface. Extended regions show geometrically viable jaw morphologies in high PC1 coordinates, but the lower PC1 borders show regions of self-intersecting geometry. Low PC1 regions are therefore geometrically impossible regions of morphospace [cf. (*9*, *27*)]. Other extreme areas may be geometrically infeasible in nature due to poor articulation surfaces with the skull, but this is not testable with jaw morphology alone.

### Functional performance of theoretical morphologies

**Fig. 3.**
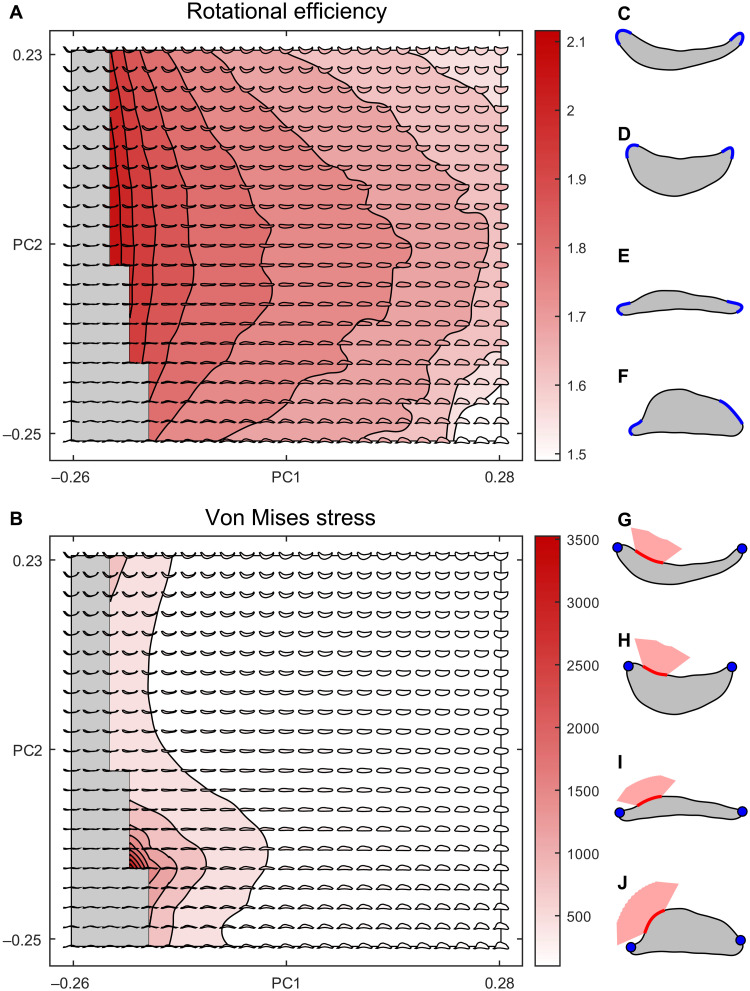
Performance surfaces generated from theoretical shapes. RE (**A**) and median VMS (**B**) performance surfaces showing the mean value from 1000 random constraint inputs. Superimposed theoretical shapes are colored on the basis of their individual performance. Gray shapes and area represent geometrically impossible shapes and morphospace. (**C** to **J**) Random inputs shown on four theoretical shapes from different grid positions for RE (C to F) (blue lines represent the boundaries of random joint and tooth placement) and VMS (G to J) (blue dots represent constraint positions, red line represents boundaries of random force placement, and red area represents boundaries of random force direction).

We conducted functional analysis on the theoretical shapes. All taxa in the dataset are aquatic and encountered obstacles imposed by aquatic feeding, such as the bow wave produced by extending jaws (*28*). Yet, they also benefit from feeding in a dense medium where suction feeding is feasible for prey capture (*14*, *29*, *30*). The first functional metric that we analyzed therefore was the RE of the jaw as a proxy for speed of the jaw opening and closing due to its role in defining the time to peak gape and therefore suction feeding performance (*5*, *29*, *31*–*33*). We defined RE as the speed of the jaw tip, given one unit of angular kinetic energy, which is dependent on the length and the moment of inertia of jaw shape (*5*). Stress resistance was the second functional metric determined, as it is a common adaptive feature tested in feeding systems to gauge resistance to the forces generated during biting (*13*, *18*). For inferring stress resistance, we used the median von Mises stress (VMS) of a finite element model subject to jaw loading: Models were fixed at a jaw hinge and anterior bite point and loaded with muscle force. To account for uncertainty in boundary condition position and orientation for the theoretical jaw shapes, we varied the locations of the jaw hinge and bite position over 5% of the total length of the jaw for the calculation of RE (Fig. 3A). Muscle force location was also varied over 5% total jaw length, and muscle force orientation was varied 45° in either direction. All theoretical shapes were analyzed with these constraints and randomized 1000 times, resulting in a total of 430,000 finite element analysis (FEA) models and 430,000 calculations of RE. To infer the performance of jaw shape only, we standardized all theoretical jaw morphologies to the same surface area. Performance surfaces were constructed from the mean (Fig. 3) and 5th and 95th percentile performance values of each functional metric (figs. S6 to S8).

The RE performance surface (Fig. 3A) shows a clear relationship between shape and speed. Geometrically viable regions at low PC1 show greater RE compared to high PC1 regions, and more intermediate PC2 values have greater RE than the extremes of PC2. The RE is well characterized by a second order polynomial surface [sum of squared estimate of errors (SSE) = 0.3619, root mean square error (RMSE) = 0.0293, coefficient of determination (*R*^2^) = 0.9280, and degrees of freedom (DOF)–adjusted *R*^2^ = 0.9272], specifically a hyperbolic paraboloid. The VMS performance surface (Fig. 3B) shows a radically different shape. Regions of low PC1 and PC2 show extremely high VMS and by inference poor performance, and the variation in VMS across the majority of the theoretical morphospace is minimal in comparison to the magnitude of the low PC1-PC2 spike in stress. This surface is not well characterized by a quadratic surface (SSE = 1.2213 × 10^7^, RMSE = 170.1191, *R*^2^ = 0.6669, and DOF-adjusted *R*^2^ = 0.6630). Log transforming the VMS data produces a clearer visualization of these results (fig. S8). Given the assumption that, all else being equal, fitness increases with decreased VMS and increased RE, speed and strength are compromised within a trade-off, as longer structures with more mass distributed toward the pivot point (low PC1 values) rotate faster and experience larger stresses than shorter structures of more homogeneous thickness (high PC1 values).

### Pareto optimality and the occupation of theoretical morphospace

**Fig. 4.**
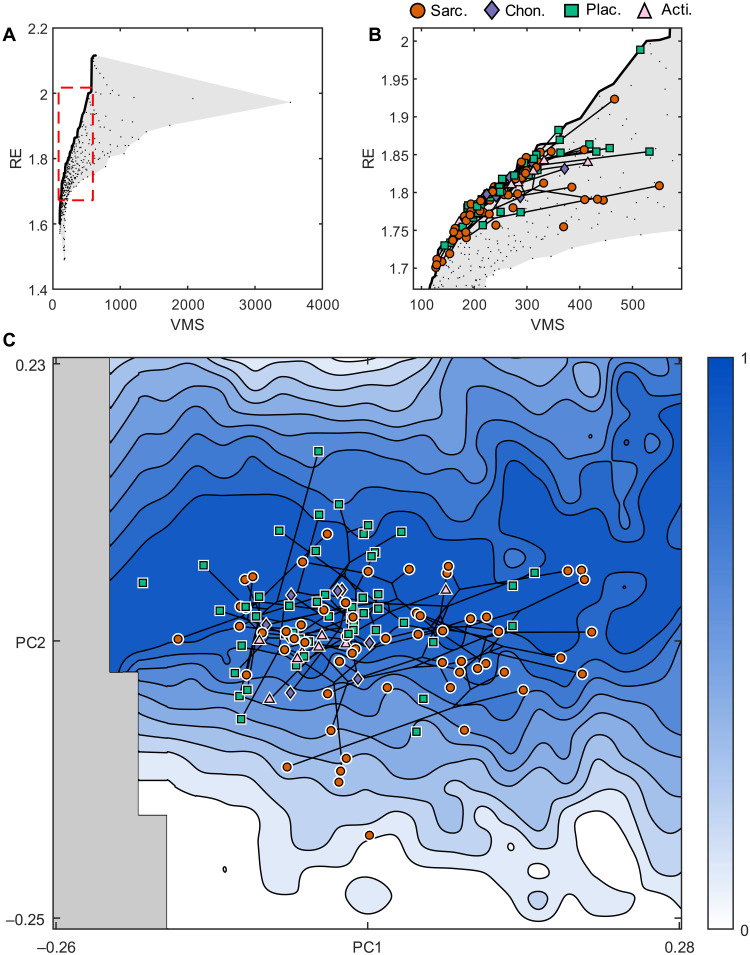
Pareto optimality and the adaptive landscape. (**A**) Performance space. Each theoretical shape is represented by a black dot, plotted at by its individual VMS and RE performance. Note the heterogeneous occupation densities. Gray area represents the region of possible solutions. Solid black line represents the Pareto front. Red dashed area represents the area shown in (**B**), a zoom of plot (A), showing extrapolated taxon performances and their phylogenetic relationships. (**C**) The adaptive landscape, with phylomorphospace superimposed. Note that only 99 of the 121 taxa are included in the phylogeny; the remainder are plotted unconnected to the phylogeny. Pareto rank represents optimality, with 0 being least optimal and 1 representing optimal (on the Pareto front). Gray region represents geometrically impossible morphospace. Legend shows symbols and colors for individual taxa from four clades: Sarcopterygii (Sarc.), Chondrichthyes and acanthodians (Chon.), Placodermi (Plac.), and Actinopterygii (Acti.).

Plotting the strength of each theoretical jaw morphology against its speed (Fig. 4A) highlights the trade-off in our chosen performance metrics and reveals that many theoretical shapes have low stress scores and intermediate RE (many-to-one mapping of form to function) (*14*). Within this “performance space,” it is possible to establish the Pareto front of theoretical shapes, i.e., those theoretical shapes in which neither metric can be improved without deteriorating performance of the other (*34*).

To establish how early jaw evolution explored this performance space, we modeled the evolution of jaw shape on a time-scaled phylogeny of early jawed vertebrates, allowing us to reconstruct mandible morphology for the ancestors of the fossil jawed vertebrates sampled, including the ancestral jawed vertebrate (fig. S9). These ancestral jaw shapes were projected into the performance landscape along with the sampled taxa (Fig. 4B). On this basis, we find that most early gnathostomes occupy the Pareto front, corroborating our prior view of the adaptive value and trade-off of the functional metrics tested. However, some taxa plot further from the optimal boundary in regions of relatively high stress and intermediate RE. Many of the jaws occupying suboptimal regions of performance space independently migrated to this region from the Pareto front and did so by decreasing strength rather than decreasing RE (Fig. 4B). This migration may be the result of another attractor in performance space caused by an additional functional metric not tested here or optimizing stress resistance becomes much less important to these taxa than optimizing RE or both.

We developed a Pareto rank algorithm that assigns each theoretical shape a value from 0 (suboptimal) to 1 (optimal) (see Methods). Plotting the Pareto rank of each theoretical shape on the *z* axis above the morphospace generates the adaptive landscape of theoretical shapes (Fig. 4C, blue scale from 0 to 1). The optimality rank of empirical taxa was extrapolated from their location on this surface (Fig. 4C, data points). The majority of early jawed vertebrates occupy a Pareto optimal region of space, with some taxa showing extension into suboptimal space. One explanation for this phenomenon may be that “suboptimal” taxa in the PC1-PC2 plane lie in higher-dimensional space that is optimal. If this were the case, then we may expect taxa further away from the PC1-PC2 plane to lie in the less optimal regions on the PC1-PC2 plane. No significant correlation between the Euclidean distance from individual taxa to the plane of theoretical morphospace and their extrapolated optimality was found (ρ = −0.0217 and *P* = 0.8127). Therefore, taxon suboptimality is unlikely to be due to shape variation that is not captured by PC1 and PC2.

### Disparity and optimality

**Fig. 5.**
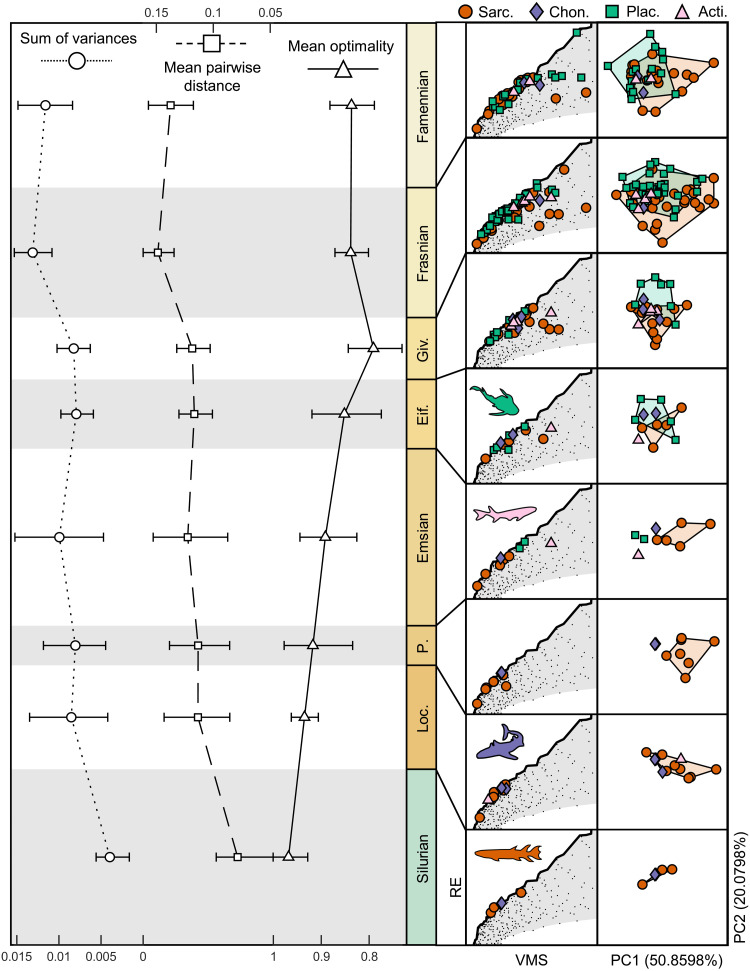
Disparity and optimality through time. Mean optimality decreases steadily through time, while the sum of variances and mean pairwise distance increase through the Devonian. White symbols and error bars represent mean and 95 confidence intervals of 10,000 bootstrap replicates. Columns on the right represent the occupation change in performance space (VMS versus RE) and morphospace (PC1 versus PC2) over time. Legend shows symbols and colors for individual taxa from four clades: Sarcopterygii (Sarc.), Chondrichthyes and acanthodians (Chon.), Placodermi (Plac.), and Actinopterygii (Acti.).

Early jawed vertebrate taxa generally occupy only optimal regions of stress and speed function, with the exception of some sarcopterygian taxa that occupy suboptimal performance regions at lower PC2 values (Fig. 4, B and C). In particular, low optimality sarcopterygian taxa include *Nesides* (0.1239), *Soederberghia* (0.3389), *Diplocercides* (0.3462), *Serenichthys* (0.3781), and *Miguashaia* (0.4067). These are coelacanths and a lungfish, suggesting some phylogenetic correlation to suboptimality. The phylomorphospace (Fig. 4C) shows that many taxa have independently evolved similar morphologies, evidencing a widespread convergence in jaw shape. A multivariate *K* statistic (*K*_mult_) (*35*) showed a weak but significant phylogenetic signal in the shape data (*K*_mult_ = 0.37425, *P* = 0.0001). Focusing on the empirical data, we measure disparity in our dataset through two metrics (sum of variances and mean pairwise distance), which were chosen due to their robusticity to sample size (*36*). Mean pairwise distance shows significant steady increase through evolutionary time, while the sum of variances shows a similar trend that is not significant (Fig. 5 and dataset S1C). However, measurements from consecutive time bins show large overlap in bootstrap confidence intervals in all metrics (Fig. 5). This shows the disconnection between patterns of morphological and functional disparity (*6*), which could be due to the “many forms, one function” nature of morphology, or the mechanical sensitivity of the system, which has been demonstrated to affect disparity measures (*14*, *37*). Mean taxon optimality for each time bin shows a steady decrease with time and has a significant negative relationship with mean pairwise distance (Fig. 5 and dataset S1D).

## DISCUSSION

We find that a large range of theoretical shapes exhibit optimal performance within a tradeoff for RE and jaw strength, particularly those with low curvature and a mass distribution that is weighted toward the jaw articulation (Fig. 4). This is because the jaws that harbor more mass close to their pivot have inherently lower rotational inertia and therefore higher RE while still maintaining large areas of mass for stress distribution. Pareto ranking characterizes the optimality of a set of solutions (in this case, theoretical morphologies) relative to one another when considering their individual performances (in this case, their strength and speed). This does not denote the adaptive value or fit of a jaw to a specific ecological role. We do not assess the relative importance of different functional metrics, as has been successful in studies of extant organisms where accurate ecological data are available (*13*, *15*–*17*, *19*). Here, we interpret the optimality of each jaw as a 2D vector of weights, **w**, which determines the adaptive value of the two dimensions of performance (strength and speed, represented in a 2D performance space; Figs. 1 and 4, A and B). We do not need to know the precise magnitude or direction of **w**, as Pareto ranking allows us to identify the range of optimal morphologies given only the quadrant that **w** occupies. In practice, this is equivalent to our assumption that higher RE is adaptive and higher VMS is maladaptive. Despite many taxa exhibiting optimal ranking for the speed-strength trade-off, we also find that there are large areas of space unoccupied by empirical taxa that are ranked highly within our system. Taxa may not explore these areas, because they exhibit dichotomous performance—the morphologies in that space perform exceptionally in one metric but very poorly in the other.

We find that the earliest gnathostome jaws, and their inferred ancestral stages, have mandibles that are optimized for a tradeoff for speed and strength, therefore supporting a predatory function. The distribution of taxa within the boundaries of our theoretical jaw morphospace demonstrates that their jaws were optimized for this tradeoff (Fig. 4 and fig. S10). Thus, optimality was achieved very early in jaw evolution, and our time-sliced performance space reveals that much of the subsequent exploration of shape space tracks the optimality front (Fig. 5). This pattern was maintained in each major clade or grade, with Placodermi, Chondrichthyes (here including acanthodians), Actinopterygii, and Sarcopterygii all exhibiting optimal jaw morphologies early in their evolution. Disparity increased as placoderms and sarcopterygians diversified into opposing regions of morphospace. Within the placoderms, many arthrodires occupy higher PC2 regions, representing a shift to stronger and less rotationally efficient morphologies. In the extreme case, *Gorgonichthys* represents a diversification into suboptimal shape space, reflecting a shift toward decreased strength while maintaining RE. Sarcopterygians diversified into shape space are also characterized by increased strength but lower speed efficiency (higher PC1 coordinates). Lungfish and actinistians independently evolved suboptimal morphologies that have characteristics comparable to *Gorgonichthys*: decreasing strength while maintaining RE. Lower strength in lungfish jaws is unexpected and perhaps inconsistent with the durophagous features of dipnoan jaws; we hypothesize that this is due to variation in the medial aspect not captured by our analysis or the loss of data to higher PC axes. Despite exploring different extremes of morphospace, Sarcopterygii and Placodermi exhibit functional convergence. Chondrichthyans and actinopterygians remained confined to their initial range of morphologies within Pareto optimal space.

Our results are compatible with the hypothesis that the mandibles of the earliest jawed vertebrates were optimized for prey acquisition and processing (*38*–*40*). Rather than diversifying through shape space until an optimal morphology is achieved, the early evolution of jawed vertebrates is characterized by diffusion among equally optimal but morphologically disparate jaw morphologies. It is inevitable that the known fossil record misses an initial evolutionary episode of mandibular evolution, but our phylomorphospace approach, in which we infer morphologies ancestral to those sampled, diminishes the impact of this formal possibility. Deviation from morphologies optimized for prey acquisition and processing is a feature of later phylogenetic history. While the variety of mandibular functional morphology remains static (*6*), mandibular shape disparity increases through time (Fig. 5), made possible by the evolutionary discovery of morphologically divergent mandibles of the same optimality (Fig. 4A). Despite the repeated evolution of Pareto optimal morphologies as taxa go extinct and new taxa originate, average jaw optimality decreases with increasing time and disparity.

Decreasing optimality is caused largely by the independent evolution of taxa with jaws that are weaker but otherwise maintain RE. The most optimal jaw shapes occupy a band of morphospace across PC1 coincident with largely straight jaws. Shifting into negative or positive PC2 space results in suboptimal jaws that are convex or concave, respectively. Thus, the shift from the Pareto front may be related to the repeated evolution of jaws that are concave or convex and therefore not optimized for a strength-speed trade-off. This may be due to the introduction of an ecological factor that may have changed the importance of the adaptive criterion of stress resistance or added new attractors in performance space. Examples of ecological change might be the emergence of ram feeding, lunge feeding, and other planktivorous strategies (*41*, *42*) that are unlikely to require strong jaws but still rely on efficient jaw movement. Durophagy is another new feeding mode established within this evolutionary episode, but adaptation to hard food diets has often been associated with stronger jaw morphologies (*3*, *18*, *43*). Decreasing optimality within our system is more likely driven by planktivorous feeding modes than durophagy, as the former is consistent with the pattern of decreasing strength and constant RE in suboptimal jaws. However, this pattern may be an artefact of our 2D approach, since, unlike the majority of jaws sampled for our study, durophagous jaws have a complex 3D morphology. Similarly, the perceived shift away from Pareto optimal morphologies may also reflect the evolution of new musculature systems that redefine the loading conditions of the biting mandible (*44*–*46*), resulting in contrasting stress patterns and magnitudes being subject to selection. Size may be another important factor, as larger (longer) jaws in our dataset occur at the limits of variation within the empirical data (fig. S11). However, most of the size variation is evenly spread across morphospace. Alternatively, functional constraints on jaw morphology may have weakened through time or weakened with increasing disparity, as increasingly less of optimal morphospace remains to be discovered.

In any instance, our results reveal a pattern of increasing mandibular morphological disparity with clade diversification despite stasis in the evolution of jaw functional disparity (*6*). This difference reflects the interrogative nature of our integrative theoretical morphology, functional optimality, and phylomorphospace approach to analyzing the evolution of form and function. It reveals that, although there may be a rapid rise to stasis in variance of phenotypic characters linked to function, this does not equate to stasis in phenotypic evolution, since our analyses demonstrate disparate morphologies that are equally Pareto optimal. The early evolution of jawed vertebrates is therefore characterized by the progressive exploration and convergence upon functionally equivalent phenotypes.

Following strong functional constraint early in gnathostome jaw evolution, increasing morphological disparity coupled with decreasing optimality suggests that the landscape of optimality roughens with the emergence of new, functionally relevant anatomical innovations and that functional constraints (strength and speed) on morphospace occupation may have relaxed over time. This provides broader insight into questions surrounding the evolution of disparity through clade history, supporting a view that morphospace may not necessarily become saturated after an early burst in disparity (*47*, *48*). Rather, disparity can continue to increase, as patterns of functional optimality are rearranged and complexified within theoretical morphospace, reflecting the adaptation of taxa to different functions and trade-offs in response to their changing environment (exploitation of new prey or of old prey in a new way). Our results provide not only a further example of continuous disparity increase through clade history but also a causal mechanism for disparity accumulation: many divergent morphologies initially converging and then diverging from functional optima. Our approach to investigating disparity has allowed us to reach beyond traditional qualitative assessments of functional constraints on morphospace occupation by empirical datasets to quantitative testing of functional limits of theoretically plausible forms. By focusing functional analysis on theoretical morphospace, we can test adaptive hypotheses on the evolution of morphology and the accumulation of disparity while avoiding prior assumptions of fitness.

## METHODS

### Dataset

Our dataset consists of lower jaw shapes of 121 extinct gnathostome taxa ranging in age from the late Silurian to the end of the Devonian. Data were time binned, using age data sourced from the literature and the Paleobiology Database (dataset S1A), to each of the Devonian stages, while Silurian taxa were grouped into one time bin representing the late Silurian due to the low data availability. Taxa that existed in more than one bin were ranged through and counted in all intermediate time bins. The dataset was also split into four clades: Sarcopterygii (*N* = 57), Placodermi (*N* = 48), Chondrichthyes (including acanthodians) (*N* = 8), and Actinopterygii (*N* = 8) (dataset S1A). Images of lower jaws were sourced from the literature using photographs of lateral shape, reconstructions, and, where available, computed tomography scans. The lateral 2D shape of the jaw was chosen because of its prevalence in the fossil record, in figures within journal articles and books, and its previous use in the literature as a model for functional processes (*5*–*7*, *46*, *49*) due to many jaw shapes approximating a 2D planar shape. However, it is noted that the jaw shape in these taxa does have some 3D variation, and this will affect disparity and functional metrics (*50*).

### Shape analysis

A further advantage to analyzing 2D jaw shape is that 2D filled polygons (such as the lateral jaw silhouette) can be characterized with zero converging error by curves with distinct functions, output from Fourier deconstructions (*11*, *51*). Specifically, EFA was the chosen method for morphometrics due to its shape characterization and reconstruction ability (*8*, *11*, *52*, *53*). Sensitivity tests of input outline data informed a decision to characterize the data with 600 outline landmarks, from which 12 EFA harmonics were generated. Six hundred were chosen, as it was the maximum number possible across all image files, and it was considerably beyond the point of convergence (see the Supplementary Materials). Size and rotation variation of each curve was eliminated (*8*), with the goal of characterizing the jaw shape alone. This resulted in a dataset of 45 continuous characters (4 × 12 − 3). From these data, a PCA was used to build a morphospace of maximum variation (*11*) within empirical shape data (Figs. 1 and 2A).

The empirical 2D shapes are generated via a mathematical formula that plots elliptic harmonics. Changing the input parameters of this formula generates a proportional change in 2D shape. We exploited this process to generate a grid of parameterized theoretical jaw shapes (Figs. 1 and 2B). We produced a 23-by-21 grid of 483 of evenly spaced theoretical jaw shapes, which was plotted across the PC1-PC2 morphospace (Fig. 1). These 483 jaws were designed to cover the space occupied by empirical data plus an extended border range of 20% to infer the patterns at the extremes of unoccupied morphospace. Of these 483 meshes, 53 self-intersecting loops were omitted from any functional analysis, leaving 430 testable meshes. Self-intersection is an ostensibly impossible feature of any 3D structure in 2D lateral view; thus, these regions were defined as a geometrically impossible space (*9*, *27*).

### Phylogeny

The phylogenetic tree used in all analyses was an informal supertree assembled from a multitude of literature sources (data S1A, fig. S9) (*54*–*72*). The tree included 99 of the 121 taxa from the analysis that could be found in phylogenies from the literature and was dated using the “equal” method of the function bin_timePaleoPhy in the R package paleotree (*73*). The R package geomorph was used to assess the phylogenetic signal of the data and perform maximum likelihood ancestral state reconstruction (functions physignal and gm.prcomp) (*74*). These functions were used due to their applicability to coordinate data, matching the EFA output data. Each axis of each elliptical harmonic was considered as a single 2D coordinate. Ancestral states were not included in the PCA to build the morphospace; instead, they were transformed into the co-ordinate space using the PC coefficients. All R analyses were performed with R v.3.6.1.

### Functional analysis

Each of the 483 theoretical jaws was converted into meshes of 2500 triangular elements. A sensitivity test performed on 10 meshes incremented by 500 elements showed that, at 1000 random replications, a mesh density of 2500 elements was adequate for convergence in both functional metrics. We calculated RE, defined as the velocity of the tip of the jaw when rotating about the jaw hinge given one unit of energy. RE can be considered a proxy for jaw closure speed. The RE was thus calculated for each theoretical shape as the velocity of the bite point (*v*) given a rotational energy of 1 J, where

$$v=L\sqrt{\frac{2}{I}}$$

where *L* is the length of the distance between bite point and the rotational axis (jaw hinge) and *I* is the moment of inertia at the bite point, where *I* can be calculated with discrete finite elements using this formula (*5*)

$$I\approx \sum_{i=1}^{N}m_{i}{r_{i}}^{2}$$

where *m* = element mass, *r* = distance from the element center of mass to rotational axis, and *N* = the number of elements. As each theoretical jaw shape is standardized by area, their lengths still vary. This calculation uses the length of the size-standardized jaws and thus is equivalent to the length divided by the square root of jaw area. RE was first calculated taking the posterior-most node of the jaw outline with a near vertical normal as the initial jaw joint and the anterior-most node of the jaw outline with a near vertical normal as the initial bite point. Jaw joint and bite point positions were then bootstrapped over 1000 randomizations that varied the joint and bite location along 5% of the total outline length on either side of the initial placement. Mean and 95% confidence intervals were then generated for each theoretical shape. The functional landscape of mean values is reported here; see fig. S6 for 5th and 95th percentile results.

We calculated the VMS across each theoretical jaw shape mesh using a simple 2D constant strain triangle FEA algorithm in MATLAB.Each jaw was modeled as a thin plate of uniform thickness with a Young’s modulus of 2 × 10^9^ Pa and Poisson’s ratio of 0.3. VMS has been used a proxy for strength in recent functional adaptive landscape studies (*19*, *23*, *75*) and has been used for a number of years as a measure of skull strength, particularly in comparative studies. Our functional landscapes for mean, maximum, and media VMS showed little difference, so we use median VMS in this study, which is less susceptible to high stress outliers generated at constraints (*13*, *15*, *19*, *49*, *50*). The same nodes were defined as bite and jaw joint positions as for the RE calculations. The location of muscle force is then interpolated along the perimeter of the jaw between these points, initially placed one-third of the length of the jaw from the jaw joint. Again, models were tested 1000 times with pseudo-randomized input conditions, shifting the force node position by 5% of the total outline length on either side of the initial node and orienting the force direction 45° either side of the force node normal. Constraints of the jaw joint and bite position were not randomized for stress calculation due to the exponential increase in computational time that this would require and their relatively small effect on overall strain energy (*76*).

To explore the effect of raw jaw length on shape, we plotted raw jaw length against PC1 and PC2 scores for the empirical dataset and assessed the significance with a phylogenetic generalized least squares (PGLS). We find a significant relationship between raw jaw size and PC1 (*P* = 0.04129, *R*^2^ = 0.03485) and an insignificant relationship between size and PC2 (*P* = 0.4846, *R*^2^ = 0.0056). Both *R*^2^ values were low; this appears to be due to the high shape variance in smaller jaws and the lower shape variance in larger jaws. This may suggest some developmental or functional restrictions on larger jaws. We also plotted each taxon data point as a function of jaw size on a PC1-PC2 plot. We find that some of the extremes of PC space are occupied by large jaws (e.g., the placoderms *Gorgonichthys*, *Titanichthys*, and *Dunkleosteus*); however, these taxa vary in shape from short deep jaws to longer thin jaws (fig. S11).

The average median VMS value and 95% confidence intervals were then generated for each theoretical shape. As for RE, the functional landscape of mean values are reported in the main text; see figs. S7 and S8 for fifth and ninth percentile results. All functional algorithms were written in MATLAB by W.J.D. (available from Dryad, doi:10.5061/dryad.3bk3j9km8).

### Pareto optimality

Building adaptive landscapes from functional metrics often uses least squares regressions or maximum likelihood to fit a first- or second-order polynomial relationship between morphology and performance and then performance and fitness (*15*, *19*, *20*, *21*). While this gives a good approximation of fitness under certain constraints and can highlight the relative importance of each functional trait measured, this approach cannot provide a single, universal fitness metric to disparate taxa with varying ecology. Instead, each group within the dataset (e.g., each ecology) has a unique adaptive landscape. Furthermore, in this study, the VMS surface is poorly characterized by the quadratic surface required for Arnold’s fitness formulae (*21*). Here, we develop a new rank-based method of combining functional metrics into a single fitness metric, adapted from Pareto ranking algorithms (*34*, *77*). Pareto optimality has been used as a method of morphospace optimality analysis elsewhere with very promising results, although these studies operate on large-scale assumptions about the relationship between morphospace and function (*78*, *79*). We use the foundational concepts of Pareto optimality to rank morphospace location based on its functional performance.

The optimality of each theoretical morphology was ranked using a modified Goldberg Pareto ranking system (*80*). In many cases where the solutions to a problem (in this case, theoretical morphologies) experience a trade-off between *N* metrics of performance (in this case, there are two metrics: speed and strength), there exists a subset of those solutions that is Pareto optimal [Pareto optimal subset (POS)]. A solution is Pareto optimal if no other solution has better or equal performance in all metrics. We can take this concept further to generate a Pareto rank system, where the POS is assigned rank one and then removed from the sample of solutions. This allows a second POS to be found and assigned rank two. This POS is then removed from the second sample of solutions, and the process iterates until all solutions have been ranked. We develop this ranking for performance spaces with spatial occupation heterogeneity by ranking the dataset with Goldberg’s ranking (optimal ranking, *R*_O_) and then ranking it again with the optimality of each metric reversed (suboptimal ranking, *R*_S_). The rank of the solution is then calculated via this equation

$$R_{i}=\frac{R_{\text{Si}}-1}{R_{\text{Oi}}+R_{\text{Si}}-2}$$

This results in a linear rank from 0 to 1, with 1 denoting Pareto optimal (not dominated by any solution) and 0 denoting Pareto suboptimal (not dominant over any solution). Other Pareto ranking systems have been shown to be more effective in evolutionary algorithms (*81*, *82*); however, these methods are biased by the relative scales of functional metrics and the density of occupation of performance space. We opt not to use these methods to eliminate the requirement for scaling functional metrics and the bias caused by heterogeneous occupation of performance space, as equidistant theoretical forms in morphospace converge and diverge in function (Fig. 4A).

### Disparity

Both disparity metrics were measured on EFA harmonic data and bootstrapped 10,000 times. The mean optimality of each time bin was calculated by bootstrapping taxon samples 10,000 times, extrapolating optimality from the surface at individual taxon PC scores, of which the mean was calculated. Trends in each signal were tested using a Spearman rank correlation test. The disparity (sum of variances and mean pairwise distance) against optimality relationships was tested with a standard Pearson’s linear correlation test.

## Supplementary Materials

### This PDF file includes:

Figs. S1 to S12 Dataset S1 References

## Notes

View/request a protocol for this paper from *Bio-protocol*.

## References

[1] P. C. J. Donoghue, J. N. Keating, Early vertebrate evolution. Palaeontology 57, 879–893 (2014).

[2] R. H. Denison, Feeding mechanisms of agnatha and early gnathostomes. Am. Zool. 1, 177–181 (1961).

[3] K. Dennis, R. S. Miles, New durophagous arthrodires from Gogo, Western-Australia. Zool. J. Linnean Soc. 69, 43–85 (1980).

[4] P. S. L. Anderson, M. W. Westneat, Feeding mechanics and bite force modelling of the skull of *Dunkleosteus terelli*, an ancient apex predator. Biol. Lett. 3, 76–79 (2006). 10.1098/rsbl.2006.0569 PMC2373817 17443970

[5] E. Snively, P. S. L. Anderson, M. J. Ryan, Functional and ontogenetic implications of bite stress in arthrodire placoderms. Kirtlandia 57, 53–60 (2009).

[6] P. S. L. Anderson, M. Friedman, M. D. Brazeau, E. J. Rayfield, Initial radiation of jaws demonstrated stability despite faunal and environmental change. Nature 476, 206–209 (2011). 21734660 10.1038/nature10207

[7] P. S. L. Anderson, M. Friedman, M. Ruta, Late to the table: Diversification of tetrapod mandibular biomechanics lagged behind the evolution of terrestriality. Integr. Comp. Biol. 53, 197–208 (2013). 23526337 10.1093/icb/ict006

[8] F. P. Kuhl, C. R. Giardina, Elliptic fourier features of a closed contour. Comput. Graph. Image Process. 18, 236–258 (1982).

[9] G. R. McGhee Jr., Shell form in the biconvex articulate brachiopoda: A geometric analysis. Paleobiology 6, 57–76 (1980).

[10] G. R. McGhee, *The Geometry of Evolution: Adaptive Landscapes and Theoretical Morphospaces* (Cambridge Univ. Press, 2007), pp. 200.

[11] J. J. Hill, M. N. Puttick, T. L. Stubbs, E. J. Rayfield, P. C. J. Donoghue, Evolution of jaw disparity in fishes. Palaeontology 61, 847–854 (2018).

[12] B. Figueirido, Z. J. Tseng, A. Martin-Serra, Skull shape evolution in durophagous carnivorans. Evolution 67, 1975–1993 (2013). 23815654 10.1111/evo.12059

[13] M. N. Simon, R. Brandt, T. Kohlsdorf, S. J. Arnold, Bite performance surfaces of three ecologically divergent Iguanidae lizards: Relationships with lower jaw bones. Biol. J. Linn. Soc. 127, 810–825 (2019).

[14] P. C. Wainwright, M. E. Alfaro, D. I. Bolnick, C. D. Hulsey, Many-to-one mapping of form to function: A general principle in organismal design? Integr. Comp. Biol. 45, 256–262 (2005). 21676769 10.1093/icb/45.2.256

[15] P. D. Polly, C. T. Stayton, E. R. Dumont, S. E. Pierce, E. J. Rayfield, K. D. Angielczyk, Combining geometric morphometrics and finite element analysis with evolutionary modeling: Towards a synthesis. J. Vertebr. Paleontol. 36, e1111225 (2016).

[16] C. T. Stayton, Performance in three shell functions predicts the phenotypic distribution of hard-shelled turtles. Evolution 73, 720–734 (2019). 30820948 10.1111/evo.13709

[17] C. T. Stayton, L. F. O’Connor, N. M. Nisivoccia, The influence of multiple functional demands on morphological diversification: A test on turtle shells. Evolution 72, 1933–1949 (2018). 30039566 10.1111/evo.13561

[18] Z. J. Tseng, Testing adaptive hypotheses of convergence with functional landscapes: A case study of bone-cracking hypercarnivores. PLOS ONE 8, e65305 (2013). 23734244 10.1371/journal.pone.0065305 PMC3667121

[19] B. V. Dickson, S. E. Pierce, Functional performance of turtle humerus shape across an ecological adaptive landscape. Evolution 73, 1265–1277 (2019). 31008517 10.1111/evo.13747

[20] S. J. Arnold, Morphology, performance and fitness. Am. Zool. 23, 347–361 (1983).

[21] S. J. Arnold, Performance surfaces and adaptive landscapes. Integr. Comp. Biol. 43, 367–375 (2003). 21680445 10.1093/icb/43.3.367

[22] C. H. Martin, P. C. Wainwright, Multiple fitness peaks on the adaptive landscape drive adaptive radiation in the wild. Science 339, 208–211 (2013). 23307743 10.1126/science.1227710

[23] B. V. Dickson, J. A. Clack, T. R. Smithson, S. E. Pierce, Functional adaptive landscapes predict terrestrial capacity at the origin of limbs. Nature 589, 242–245 (2021). 33239789 10.1038/s41586-020-2974-5

[24] D. M. Raup, Computer as aid in describing form in gastropod shells. Science 138, 150–152 (1962). 17818401 10.1126/science.138.3537.150

[25] D. M. Raup, A. Michelson, Theoretical morphology of coiled shell. Science 147, 1294–1295 (1965). 17790826 10.1126/science.147.3663.1294

[26] D. M. Raup, Geometry of coiling in gastropods. Proc. Natl. Acad. Sci. U.S.A. 47, 602–609 (1961). 16578506 10.1073/pnas.47.4.602 PMC221494

[27] D. M. Raup, Geometric analysis of shell coiling in ammonoids. J. Paleontol. 41, 43–65 (1967).

[28] S. Van Wassenbergh, J. Brecko, P. Aerts, I. Stouten, G. Vanheusden, A. Camps, R. Van Damme, A. Herrel, Hydrodynamic constraints on prey-capture performance in forward-striking snakes. J. R. Soc. Interface 7, 773–785 (2010). 19828500 10.1098/rsif.2009.0385 PMC2874232

[29] S. W. Day, T. E. Higham, A. Y. Cheer, P. C. Wainwright, Spatial and temporal patterns of water flow generated by suction-feeding bluegill sunfishLepomis macrochirusresolved by particle image velocimetry. J. Exp. Biol. 208, 2661–2671 (2005). 16000536 10.1242/jeb.01708

[30] P. C. Wainwright, M. D. McGee, S. J. Longo, L. P. Hernandez, Origins, innovations, and diversification of suction feeding in vertebrates. Integr. Comp. Biol. 55, 134–145 (2015). 25920508 10.1093/icb/icv026

[31] S. Van Wassenbergh, S. W. Day, L. P. Hernandez, T. E. Higham, T. Skorczewski, Suction power output and the inertial cost of rotating the neurocranium to generate suction in fish. J. Theor. Biol. 372, 159–167 (2015). 25769945 10.1016/j.jtbi.2015.03.001

[32] S. Van Wassenbergh, P. Aerts, D. Adriaens, A. Herrel, A dynamic model of mouth closing movements in clariid catfishes: The role of enlarged jaw adductors. J. Theor. Biol. 234, 49–65 (2005). 15721035 10.1016/j.jtbi.2004.11.007

[33] C. P. Kenaley, Exploring feeding behaviour in deep-sea dragonfishes (Teleostei: Stomiidae): Jaw biomechanics and functional significance of a loosejaw. Biol. J. Linn. Soc. 106, 224–240 (2012).

[34] J. F. V. Vincent, The trade-off: A central concept for biomimetics. Bioinspir. Biomim. Nanobiomaterials 6, 67–76 (2017).

[35] D. C. Adams, A generalized K statistic for estimating phylogenetic signal from shape and other high-dimensional multivariate data. Syst. Biol. 63, 685–697 (2014). 24789073 10.1093/sysbio/syu030

[36] C. N. Ciampaglio, M. Kemp, D. W. McShea, Detecting changes in morphospace occupation patterns in the fossil record: Characterization and analysis of measures of disparity. Paleobiology 27, 695–715 (2001).

[37] M. M. Munoz, Y. Hu, P. S. L. Anderson, S. N. Patek, Strong biomechanical relationships bias the tempo and mode of morphological evolution. eLife 7, (2018). 10.7554/eLife.37621 PMC6133543 30091704

[38] C. Gans, R. G. Northcutt, Neural crest and the origin of vertebrates: A new head. Science 220, 268–273 (1983). 17732898 10.1126/science.220.4594.268

[39] R. G. Northcutt, Evolution of the optic tectum in ray-finned fishes, in *Fish Neurobiology 2. Higher Brain Areas and Functions,* R. E. Davis, R. G. Northcutt, Eds. (The University of Michigan Press, 1983), vol. 2, pp. 1–42.

[40] C. Gans, Stages in the origin of vertebrates: Analysis by means of scenarios. Biol. Rev. 64, 221–268 (1989). 2675997 10.1111/j.1469-185x.1989.tb00471.x

[41] L. A. Ferry, E. M. Paig-Tran, A. C. Gibb, Suction, ram, and biting: Deviations and limitations to the capture of aquatic prey. Integr. Comp. Biol. 55, 97–109 (2015). 25980566 10.1093/icb/icv028

[42] S. J. Coatham, J. Vinther, E. J. Rayfield, C. Klug, Was the Devonian placoderm *Titanichthysa* suspension feeder? R. Soc. Open Sci. 7, 200272 (2020). 32537223 10.1098/rsos.200272 PMC7277245

[43] A. M. Herbert, P. J. Motta, Biomechanics of the jaw of the durophagous bonnethead shark. Zoology (Jena) 129, 54–58 (2018). 30170748 10.1016/j.zool.2018.07.001

[44] J. Mallatt, Ventilation and the origin of jawed vertebrates: A new mouth. Zool. J. Linnean Soc. 117, 329–404 (1996).

[45] T. Miyashita, Fishing for jaws in early vertebrate evolution: A new hypothesis of mandibular confinement. Biol. Rev. 91, 611–657 (2016). 25899041 10.1111/brv.12187

[46] P. S. L. Anderson, Cranial muscle homology across modern gnathostomes. Biol. J. Linn. Soc. 94, 195–216 (2008).

[47] M. Hughes, S. Gerber, M. A. Wills, Clades reach highest morphological disparity early in their evolution. Proc. Natl. Acad. Sci. U.S.A. 110, 13875–13879 (2013). 23884651 10.1073/pnas.1302642110 PMC3752257

[48] L. J. Harmon, J. B. Losos, T. J. Davies, R. G. Gillespie, J. L. Gittleman, W. Bryan Jennings, K. H. Kozak, M. A. McPeek, F. Moreno-Roark, T. J. Near, A. Purvis, R. E. Ricklefs, D. Schluter, J. A. Schulte Ii, O. Seehausen, B. L. Sidlauskas, O. Torres-Carvajal, J. T. Weir, A. O. Mooers, Early bursts of body size and shape evolution are rare in comparative data. Evolution 64, 2385–2396 (2010). 20455932 10.1111/j.1558-5646.2010.01025.x

[49] J. Marce-Nogue, T. A. Puschel, A. Daasch, T. M. Kaiser, Broad-scale morpho-functional traits of the mandible suggest no hard food adaptation in the hominin lineage. Sci. Rep. 10, 6793 (2020). 32322020 10.1038/s41598-020-63739-5 PMC7176708

[50] N. M. Morales-Garcia, T. D. Burgess, J. J. Hill, P. G. Gill, E. J. Rayfield, The use of extruded finite-element models as a novel alternative to tomography-based models: A case study using early mammal jaws. J. R. Soc. Interface 16, 20190674 (2019). 31822222 10.1098/rsif.2019.0674 PMC6936041

[51] M. Ruta, J. Krieger, K. D. Angielczyk, M. A. Wills, The evolution of the tetrapod humerus: Morphometrics, disparity, and evolutionary rates. Earth Environ. Sci. Trans. R. Soc. Edinb. 109, 351–369 (2018).

[52] J. M. Carlo, M. S. Barbeitos, H. R. Lasker, Quantifying complex shapes: Elliptical fourier analysis of octocoral sclerites. Biol. Bull. 220, 224–237 (2011). 21712230 10.1086/BBLv220n3p224

[53] F. Márquez, J. Robledo, G. E. Peñaloza, S. Van der Molen, Use of different geometric morphometrics tools for the discrimination of phenotypic stocks of the striped clam *Ameghinomya antiqua* (Veneridae) in north Patagonia, Argentina. Fish. Res. 101, 127–131 (2010).

[54] T. J. Challands, T. R. Smithson, J. A. Clack, C. E. Bennett, J. E. A. Marshall, S. M. Wallace-Johnson, H. Hill, A lungfish survivor of the end-Devonian extinction and an Early Carboniferous dipnoan radiation. J. Syst. Palaeontol. 17, 1825–1846 (2019).

[55] L. Frey, M. Coates, M. Ginter, V. Hairapetian, M. Rucklin, I. Jerjen, C. Klug, The early elasmobranch *Phoebodus*: Phylogenetic relationships, ecomorphology and a new time-scale for shark evolution. Proc. Biol. Sci. 286, 20191336 (2019). 31575362 10.1098/rspb.2019.1336 PMC6790773

[56] A. M. Clement, B. King, S. Giles, B. Choo, P. E. Ahlberg, G. C. Young, J. A. Long, Neurocranial anatomy of an enigmatic Early Devonian fish sheds light on early osteichthyan evolution. eLife 7, e34349 (2018). 29807569 10.7554/eLife.34349 PMC5973833

[57] J. A. Clack, T. J. Challands, T. R. Smithson, K. Z. Smithson, Newly recognized Famennian lungfishes from East Greenland reveal tooth plate diversity and blur the Devonian–Carboniferous boundary. Pap. Palaeontol. 5, 261–279 (2019).

[58] B. Choo, J. Lu, S. Giles, K. Trinajstic, J. A. Long, A. Smith, A new actinopterygian from the Late Devonian Gogo Formation, Western Australia. Pap. Palaeontol. 5, 343–363 (2019).

[59] J. D. Pardo, M. Szostakiwskyj, P. E. Ahlberg, J. S. Anderson, Hidden morphological diversity among early tetrapods. Nature 546, 642–645 (2017). 28636600 10.1038/nature22966

[60] N. Takezaki, H. Nishihara, Support for lungfish as the closest relative of tetrapods by using slowly evolving ray-finned fish as the outgroup. Genome Biol. Evol. 9, 93–101 (2017). 28082606 10.1093/gbe/evw288 PMC5381532

[61] A. Kemp, L. Cavin, G. Guinot, Evolutionary history of lungfishes with a new phylogeny of post-Devonian genera. Palaeogeogr. Palaeoclimatol. Palaeoecol. 471, 209–219 (2017).

[62] B. King, T. Qiao, M. S. Y. Lee, M. Zhu, J. A. Long, Bayesian morphological clock methods resurrect placoderm monophyly and reveal rapid early evolution in jawed vertebrates. Syst. Biol. 66, 499–516 (2017). 27920231 10.1093/sysbio/syw107

[63] M. I. Coates, J. A. Finarelli, I. J. Sansom, P. S. Andreev, K. E. Criswell, K. Tietjen, M. L. Rivers, P. J. La Riviere, An early chondrichthyan and the evolutionary assembly of a shark body plan. Proc. Biol. Sci. 285, 20172418 (2018). 29298937 10.1098/rspb.2017.2418 PMC5784200

[64] M. Zhu, P. E. Ahlberg, Z. Pan, Y. Zhu, T. Qiao, W. Zhao, L. Jia, J. Lu, A Silurian maxillate placoderm illuminates jaw evolution. Science 354, 334–336 (2016). 27846567 10.1126/science.aah3764

[65] S. Giles, M. Friedman, M. D. Brazeau, Osteichthyan-like cranial conditions in an Early Devonian stem gnathostome. Nature 520, 82–85 (2015). 25581798 10.1038/nature14065 PMC5536226

[66] M. D. Brazeau, M. Friedman, The origin and early phylogenetic history of jawed vertebrates. Nature 520, 490–497 (2015). 25903631 10.1038/nature14438 PMC4648279

[67] V. Dupret, S. Sanchez, D. Goujet, P. Tafforeau, P. E. Ahlberg, A primitive placoderm sheds light on the origin of the jawed vertebrate face. Nature 507, 500–503 (2014). 24522530 10.1038/nature12980

[68] B. Choo, Revision of the actinopterygian genus *Mimipiscis* (=*Mimia*) from the Upper Devonian Gogo Formation of Western Australia and the interrelationships of the early Actinopterygii. Earth Environ. Sci. Trans. R. Soc. Edinb. 102, 77–104 (2012).

[69] K. Trinajstic, K. Dennis-Bryan, Phenotypic plasticity, polymorphism and phylogeny within placoderms. Acta Zool. 90, 83–102 (2009).

[70] M. Zhu, W. Zhao, L. Jia, J. Lu, T. Qiao, Q. Qu, The oldest articulated osteichthyan reveals mosaic gnathostome characters. Nature 458, 469–474 (2009). 19325627 10.1038/nature07855

[71] M. D. Brazeau, The braincase and jaws of a Devonian “acanthodian” and modern gnathostome origins. Nature 457, 305–308 (2009). 19148098 10.1038/nature07436

[72] J. A. Long, B. Choo, G. C. Young, A new basal actinopterygian fish from the Middle Devonian Aztec Siltstone of Antarctica. Antarct. Sci. 20, 393–412 (2008).

[73] D. W. Bapst, paleotree: An R package for paleontological and phylogenetic analyses of evolution. Methods Ecol. Evol. 3, 803–807 (2012).

[74] D. C. Adams, E. Otárola-Castillo, E. Paradis, geomorph: An R package for the collection and analysis of geometric morphometric shape data. Methods Ecol. Evol. 4, 393–399 (2013).

[75] P. D. Polly, Functional tradeoffs carry phenotypes across the valley of the shadow of death. Integr. Comp. Biol. 60, 1268–1282 (2020). 32592482 10.1093/icb/icaa092

[76] Z. J. Tseng, J. L. McNitt-Gray, H. Flashner, X. Wang, R. Enciso, Model sensitivity and use of the comparative finite element method in mammalian jaw mechanics: Mandible performance in the gray wolf. PLOS ONE 6, e19171 (2011). 21559475 10.1371/journal.pone.0019171 PMC3084775

[77] D. Adriaens, A. Lakhtakia, R. J. Martín-Palma, M. Knez, paper presented at the Bioinspiration, Biomimetics, and Bioreplication IX, 2019.

[78] O. Shoval, H. Sheftel, G. Shinar, Y. Hart, O. Ramote, A. Mayo, E. Dekel, K. Kavanagh, U. Alon, Evolutionary trade-offs, pareto optimality, and the geometry of phenotype space. Science 336, 1157–1160 (2012). 22539553 10.1126/science.1217405

[79] A. Tendler, A. Mayo, U. Alon, Evolutionary tradeoffs, Pareto optimality and the morphology of ammonite shells. BMC Syst. Biol. 9, 12 (2015). 25884468 10.1186/s12918-015-0149-z PMC4404009

[80] D. E. Goldberg, *Genetic Algorithms in Search, Optimization and Machine Learning* (Addison-Wesley Longman Publishing Co. Inc., 1989).

[81] C. M. Fonseca, P. J. Fleming, Multiobjective optimization and multiple constraint handling with evolutionary algorithms–part I: A unified formulation. IEEE Trans. Syst. Man Cybern. 1 Syst. Humans 28, 26–37 (1998).

[82] I. Alberto, C. Azcarate, F. Mallor, P. M. Mateo, Multiobjective evolutionary algorithms. Pareto rankings. Monografias del Semin. Matem. García de Galdeano 27, 27–35 (2003).

[83] P. E. Ahlberg, J. A. Clack, Lower jaws, lower tetrapods–A review based on the Devonian genus *Acanthostega*. Trans. R. Soc. Edinb. Earth Sci. 89, 11–46 (1998).

[84] M. Zhu, X. Yu, Lower jaw character transitions among major sarcopterygian groups–A survey based on new materials from Yunnan, China, in *Recent Advances in the Origin and Early Radiation of Vertebrates*, G. Arratia, M. V. H. Wilson, R. Cloutier, Eds. (Pfeil, 2004), pp. 271–286.

[85] K. S. W. Campbell, R. E. Barwick, A new tooth-plated dipnoan from the Upper Devonian Gogo Formation and its relationships. Mem. Queensl. Mus. 42, 403–437 (1998).

[86] A. Heintz, Fischreste aus dem Unterperm Norwegens. Norsk Geol. Tidsskr. 14, 176–194 (1934).

[87] J. Lu, M. Zhu, An Early Devonian (Pragian) sarcopterygian from Zhaotong, Yunnan, China. Vertebrata PalAsiatica 46, 161 (2008).

[88] H. Jessen, Weitere Fischreste aus dem Oberen Plattenkalk der Bergisch-Gladbach-Paffrather Mulde (Oberdevon, Rheinisches Schiefergebirge). Palaeontographica Abt. A 143, 159–187 (1973).

[89] J. A. Long, Cranial anatomy of two new late Devonian lungfishes (Pisces: Dipnoi) from Mount Howitt, Victoria. Rec. Aust. Mus. 44, 299–318 (1992).

[90] M. Rücklin, P. C. Donoghue, Z. Johanson, K. Trinajstic, F. Marone, M. Stampanoni, Development of teeth and jaws in the earliest jawed vertebrates. Nature 491, 748–751 (2012). 23075852 10.1038/nature11555

[91] R. K. Carr, Placoderm diversity and evolution. Bull. Mus. Natl. Hist. Nat. Paris 17, 85–125 (1995).

[92] D. H. Dunkle, A new genus and species of arthrodiran fish from the Upper Devonian Cleveland Shale. Sci. Publ. Cleve. Mus. Nat. Hist. 8, 103–117 (1947).

[93] K. S. W. Campbell, R. E. Barwick, T. J. Senden, Evolution of dipnoans (lungfish) in the Early Devonian of southeastern Australia. Alcheringa 33, 59–78 (2009).

[94] J. E. Harris, The neurocranium and jaws of *Cladoselache*. Sci. Publ. Cleve. Mus. Nat. Hist. 8, 7–12 (1938).

[95] A. Heintz, Revision of the structure of *Coccosteus decipiens* Ag. Norsk Geol. Tidsskr. 12, 291–313 (1931).

[96] L. Hussakof, W. L. Bryant, Catalogue of the fossil fishes in the museum of the Buffalo Society of Natural Sciences. Buffalo Soc. Nat. Hist. Sci. Bull. 12, 1–346 (1919).

[97] H. G. Johnson, D. K. Elliott, A new ptyctodont (Placodermi) from the Upper Devonian Martin Formation of northern Arizona, and an analysis of ptyctodont phylogeny. J. Paleontol. 70, 994–1003 (1996).

[98] E. B. Daeschler, Early tetrapod jaws from the Late Devonian of Pennsylvania, USA. J. Paleontol. 74, 301–308 (2000).

[99] M. M. Smith, M.-M. Chang, The dentition of *Diabolepis speratus* Chang and Yu, with further consideration of its relationships and the primitive dipnoan dentition. J. Vertebr. Paleontol. 10, 420–433 (1990).

[100] M. Friedman, *Styloichthys* as the oldest coelacanth: Implications for early osteichthyan interrelationships. J. Syst. Palaeontol. 5, 289–343 (2007).

[101] B. Young, R. L. Dunstone, T. J. Senden, G. C. Young, A gigantic sarcopterygian (tetrapodomorph lobe-finned fish) from the upper Devonian of Gondwana (Eden, New South Wales, Australia). PLOS ONE 8, e53871 (2013). 23483884 10.1371/journal.pone.0053871 PMC3590215

[102] J. A. Clack, *Gaining Ground: The origin and Evolution of Tetrapods* (Indiana Univ. Press, 2012).

[103] J. A. Long, A new genus of fossil coelacanth (Osteichthyes: Coelacanthiformes) from the Middle Devonian of southeastern Australia. Rec. West. Aust. Mus. 57, 37–53 (1999).

[104] J. A. Long, R. E. Barwick, K. S. W. Campbell, Osteology and functional morphology of the osteolepiform fish *Gogonasus andrewsae* Long, 1985, from the Upper Devonian Gogo Formation, Western Australia. Rec. West. Aust. Mus. 53, 1–89 (1997).

[105] B. Choo, J. A. Long, K. Trinajstic, A new genus and species of basal actinopterygian fish from the Upper Devonian Gogo Formation of Western Australia. Acta Zool. 90, 194–210 (2009).

[106] P. S. L. Anderson, Shape variation between arthrodire morphotypes indicates possible feeding niches. J. Vertebr. Paleontol. 28, 961–969 (2008).

[107] Z. Gorizdro-Kulczycka, Dwudyszne ryby dewońskie Gór Swiętokrzyskich. Acta Geol. Pol. 1, 53–105 (1950).

[108] J. P. Downs, *Holoptychius bergmanni* sp. nov.(Sarcopterygii, Porolepiformes) from the Upper Devonian of Nunavut, Canada, and a review of *Holoptychius* taxonomy. Proc. Acad. Natl. Sci. Phila. 162, 47–59 (2013).

[109] E. Mark-Kurik, The inferognathal in the Middle Devonian arthrodire *Homostius*. Lethaia 25, 173–178 (1992).

[110] J. A. Long, A new Late Devonian acanthodian fish from Mt. Howitt, Victoria, Australia, with remarks on acanthodian biogeography. Proc. R. Soc. Victoria 98, 1–17 (1986).

[111] J. A. Long, New palaeoniscoid fishes from the Late Devonian and Early Carboniferous of Victoria. Mem. Assoc. Australas. Palaeontol. 7, 1–64 (1988).

[112] E. B. Daeschler, J. P. Downs, New description and diagnosis of Hyneria lindae (Sarcopterygii, Tristichopteridae) from the Upper Devonian Catskill Formation in Pennsylvania, USA. J. Vertebr. Paleontol. 38, e1448834 (2018).

[113] E. I. White, The old red sandstone of brown clee Hill and the adjacent area II. Palaeontology, in *Bulletin of the British Museum (Natural History), Geology* [London: BM(NH), 1961], vol. 5, pp. 243–310.

[114] E. I. Vorobyeva, Morphology and nature of evolution of crossopterygian fish. Trudy Paleontologicheskogo Instituta 163, 1–239 (1977).

[115] K. Trinajstic, J. A. Long, A new genus and species of Ptyctodont (Placodermi) from the Late Devonian Gneudna Formation, Western Australia, and an analysis of Ptyctodont phylogeny. Geol. Mag. 146, 743–760 (2009).

[116] E. I. Vorobyeva, A new species of *Laccognathus* (Porolepiform Crossopterygii) from the Devonian of Latvia. Paleontol. Zhurnal 40, 312–322 (2006).

[117] J. Kulczycki, Upper devonian fishes from the holy cross mountains. Acta Palaeontol. Pol. 2, 285–380 (1957).

[118] J. A. Long, K. Trinajstic, G. C. Young, T. Senden, Live birth in the Devonian period. Nature 453, 650–652 (2008). 18509443 10.1038/nature06966

[119] M. Zhu, W. Wang, X. Yu, *Meemania eos*, a basal sarcopterygian fish from the Lower Devonian of China–Expanded description and significance, in *Morphology, Phylogeny and palaeobIogeography of Fossil Fishes,* D. K. Elliott, J. G. Maisey, X. Yu, D. Miao, Eds. (Verlag Dr Friedrich Pfeil, 2010), pp. 199–214.

[120] B. Choo, M. Zhu, W. Zhao, L. Jia, Y. Zhu, The largest Silurian vertebrate and its palaeoecological implications. Sci. Rep. 4, 5242 (2015). 10.1038/srep05242 PMC4054400 24921626

[121] P. L. Forey, P. E. Ahlberg, E. Lukševičs, I. Zupinš, A new coelacanth from the Middle Devonian of Latvia. J. Vertebr. Paleontol. 20, 243–252 (2000).

[122] N. I. Krupina, R. R. Reisz, D. Scott, The skull and tooth system of *Orlovichthys limnatis*, a Late Devonian dipnoan from Russia. Can. J. Earth Sci. 38, 1301–1311 (2001).

[123] A. M. Clement, “The anatomy, evolution and interrelationships of Devonian Dipnoans, with insights from the extant Australian lungfish, *Neoceratodus forsteri*,” thesis, Australian National University (2011).

[124] T. Ørvig, Histologic studies of ostracoderms, placoderms and fossil elasmobranchs 3. Structure and growth of gnathalia of certain arthrodires. Zool. Scr. 9, 141–159 (1980).

[125] E. Jarvik, Middle and Upper Devonian porolepiformes from East Greenland with special reference to *Glyptolepis groenlandica* n.sp. and a discussion on the structure of the head in the porolepiformes. Medd. Grønland 187, 1–307 (1972).

[126] H. L. Jessen, Lower Devonian Porolepiformes from the Canadian Arctic with special reference to *Powichthys thorsteinssoni* Jessen. Palaeontographica Abt. A 167, 180–214 (1980).

[127] G. F. Hanke, *Promesacanthus eppleri* n. gen., n. sp., a mesacanthid (Acanthodii, Acanthodiformes) from the Lower Devonian of northern Canada. Geodiversitas 30, 287–302 (2008).

[128] R. S. Miles, The acanthodian fishes of the Devonian Plattenkalk of the Paffrath Trough in the Rhineland, with an appendix containing a classification of the Acanthodii and a revision of the genus *Homalacanthus*. Arkiv Zool. 18, 147–194 (1966).

[129] R. W. Gess, M. I. Coates, Fossil juvenile coelacanths from the Devonian of South Africa shed light on the order of character acquisition in actinistians. Zool. J. Linnean Soc. 175, 360–383 (2015).

[130] D. Snitting, A redescription of the anatomy of the Late DevonianSpodichthys buetleriJarvik, 1985 (Sarcopterygii, Tetrapodomorpha) from East Greenland. J. Vertebr. Paleontol. 28, 637–655 (2008).

[131] E. B. Daeschler, N. H. Shubin, F. A. Jenkins, A Devonian tetrapod-like fish and the evolution of the tetrapod body plan. Nature 440, 757–763 (2006). 16598249 10.1038/nature04639

[132] J. Boyle, M. J. Ryan, New information onTitanichthys(Placodermi, Arthrodira) from the Cleveland Shale (Upper Devonian) of Ohio, USA. J. Paleontol. 91, 318–336 (2017).

[133] J. Lu, M. Zhu, J. A. Long, W. Zhao, T. J. Senden, L. Jia, T. Qiao, The earliest known stem-tetrapod from the Lower Devonian of China. Nat. Commun. 3, 1160 (2012). 23093197 10.1038/ncomms2170

[134] R. S. Miles, T. S. Westoll, IX.—Two new genera of coccosteid arthrodira from the Middle Old Red Sandstone of Scotland, and their Stratigraphical distribution. Trans. R. Soc. Edinburgh 65, 179–210 (1962).

[135] T. Ørvig, Some new acanthodian material from the Lower Devonian of Europe. Zool. J. Linnean Soc. 47, 131–153 (1967).

[136] J. A. Clack, P. E. Ahlberg, H. Blom, S. M. Finney, A new genus of Devonian tetrapod from North-East Greenland, with new information on the lower jaw of Ichthyostega. Palaeontology 55, 73–86 (2012).

