# Supplementary material for: Increasing morphological disparity and decreasing optimality for jaw speed and strength during the radiation of jawed vertebrates
Source: Sci Adv. 2022 Mar 18;8(11):eabl3644. doi: 10.1126/sciadv.abl3644 (PMC8932669; doi:10.1126/sciadv.abl3644)
Supplement: Supplementary file 1 — Figs. S1 to S12 Dataset S1 References [file sciadv.abl3644_sm.pdf]

Science Advances
AAAS

Supplementary Materials for

Increasing morphological disparity and decreasing optimality for jaw speed and strength during the radiation of jawed vertebrates

William J. Deakin*, Philip S. L. Anderson, Wendy den Boer, Thomas J. Smith, Jennifer J. Hill, Martin Rücklin, Philip C. J. Donoghue*, Emily J. Rayfield*

*Corresponding author. Email: wd15899@bristol.ac.uk (W.J.D.); phil.donoghue@bristol.ac.uk (P.C.J.D.); e.rayfield@bristol.ac.uk (E.J.R.)

Published 18 March 2022, *Sci. Adv.* **8**, eabl3644 (2022)
DOI: 10.1126/sciadv.abl3644

This PDF file includes:

Figs. S1 to S12
Dataset S1
References

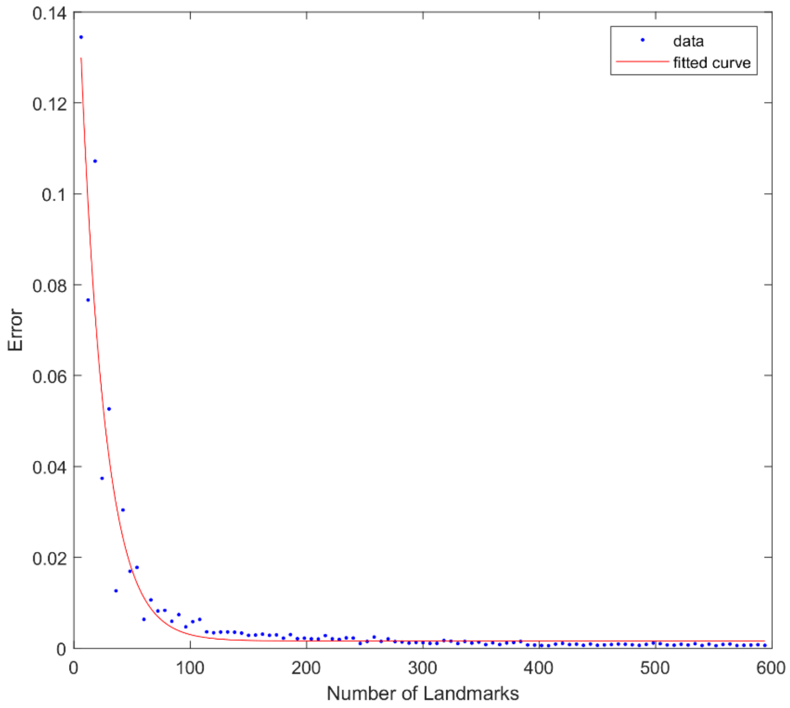

Fig S1: Landmark convergence test on *Acanthostega* outline. Optimal convergence is reached significantly before the chosen number, 600. 600 was chosen because it was the maximum possible number usable across all images, given varying image resolution, and the landmark number does not affect computation time significantly.

Morphospace

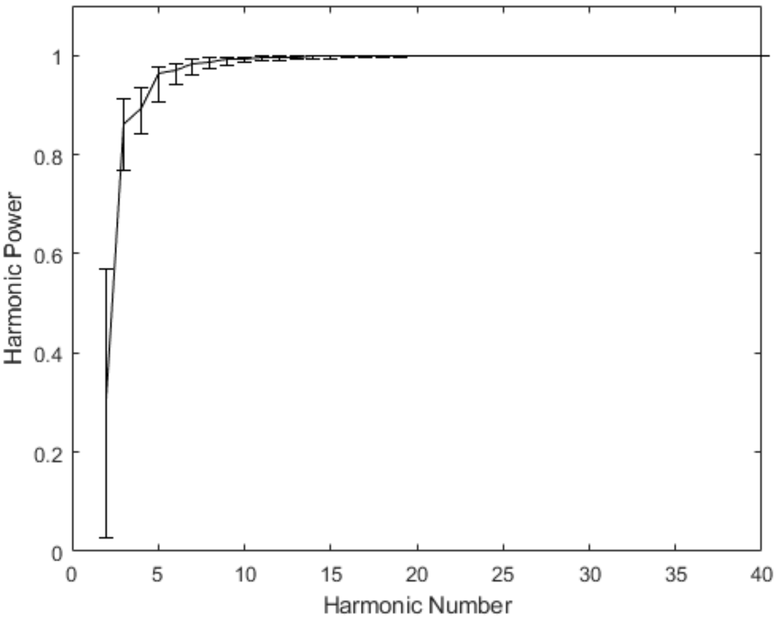

Fig S2: Harmonic power convergence across all taxa. Optimum number of harmonics was chosen as the point where the 5th percentile of taxa crosses the 0.99 power boundary (12 harmonics).

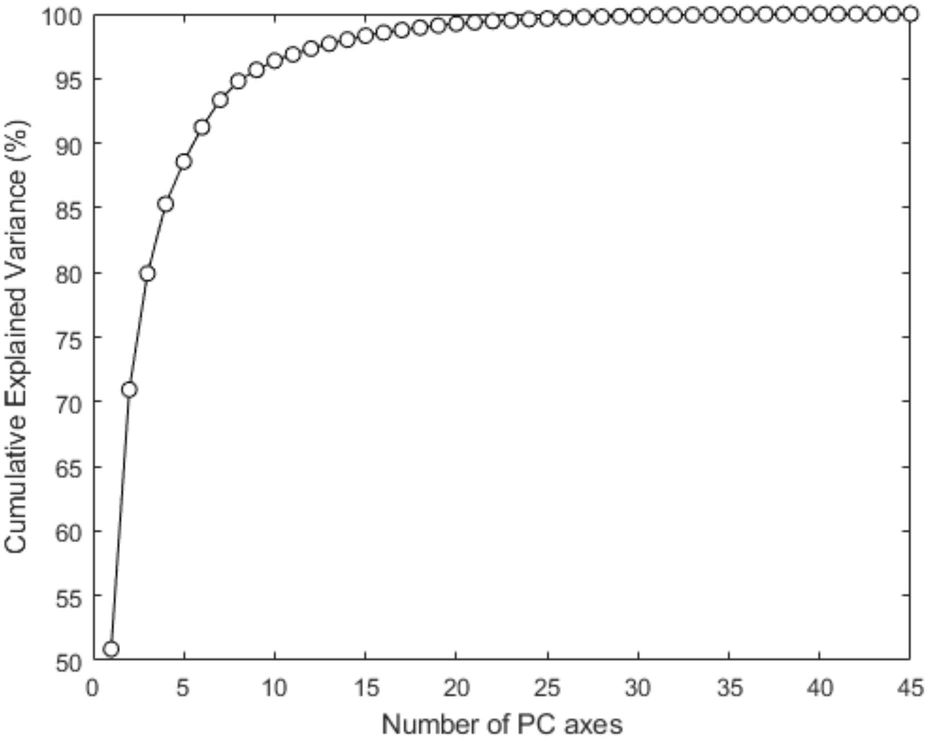

Fig S3: PCA Scree plot.

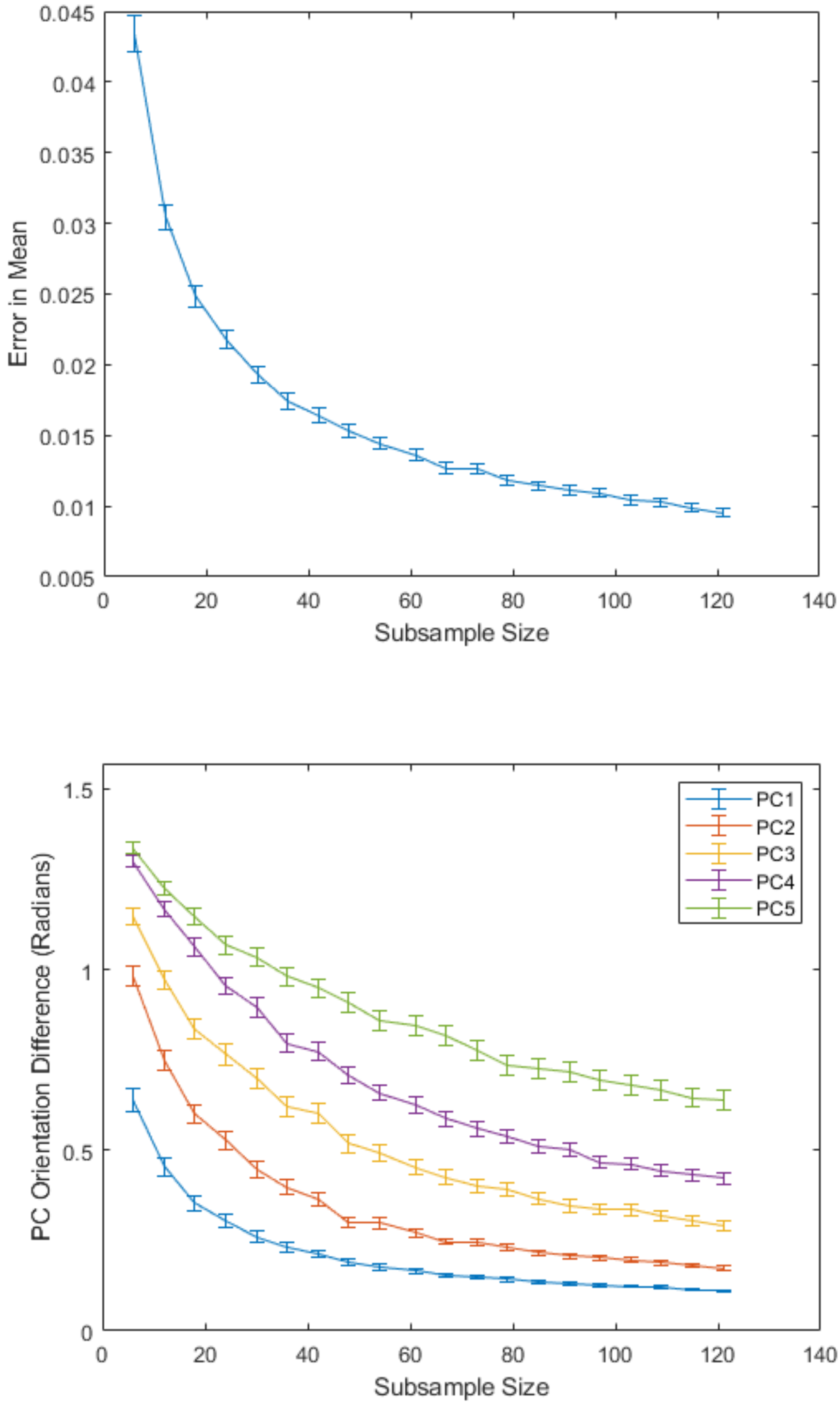

Fig S4: Sensitivity tests on theoretical morphospace at different sample sizes. Subsamples are built from bootstrapping the taxa and reperforming PCA. Top: Error in mean, Bottom: Error in orientation of the first 5 PC axes.

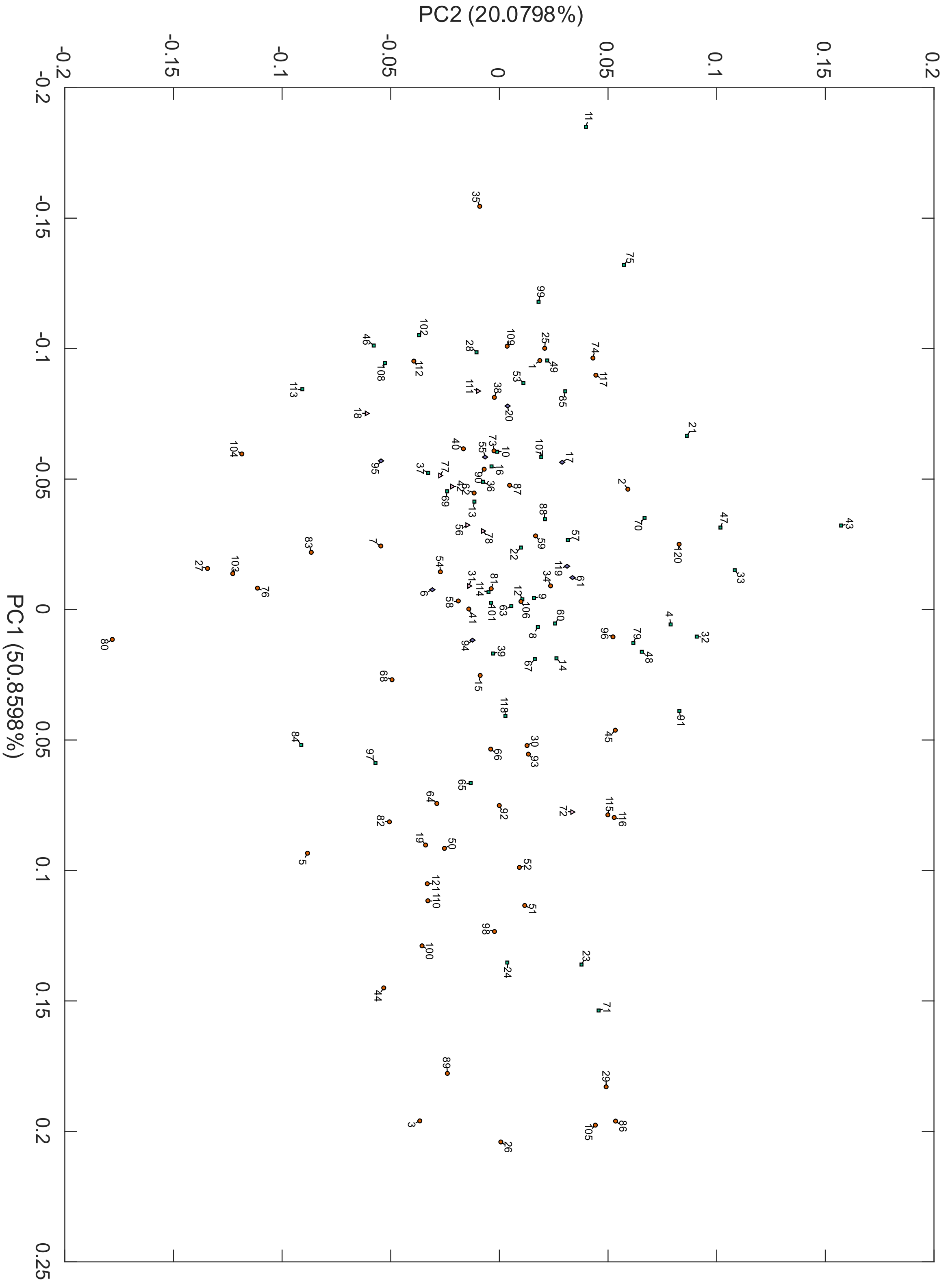

Fig S5: PC axes 1 and 2 with individual taxa labelled. Numbers represent taxon index (See Dataset S1A)

Functional Performance

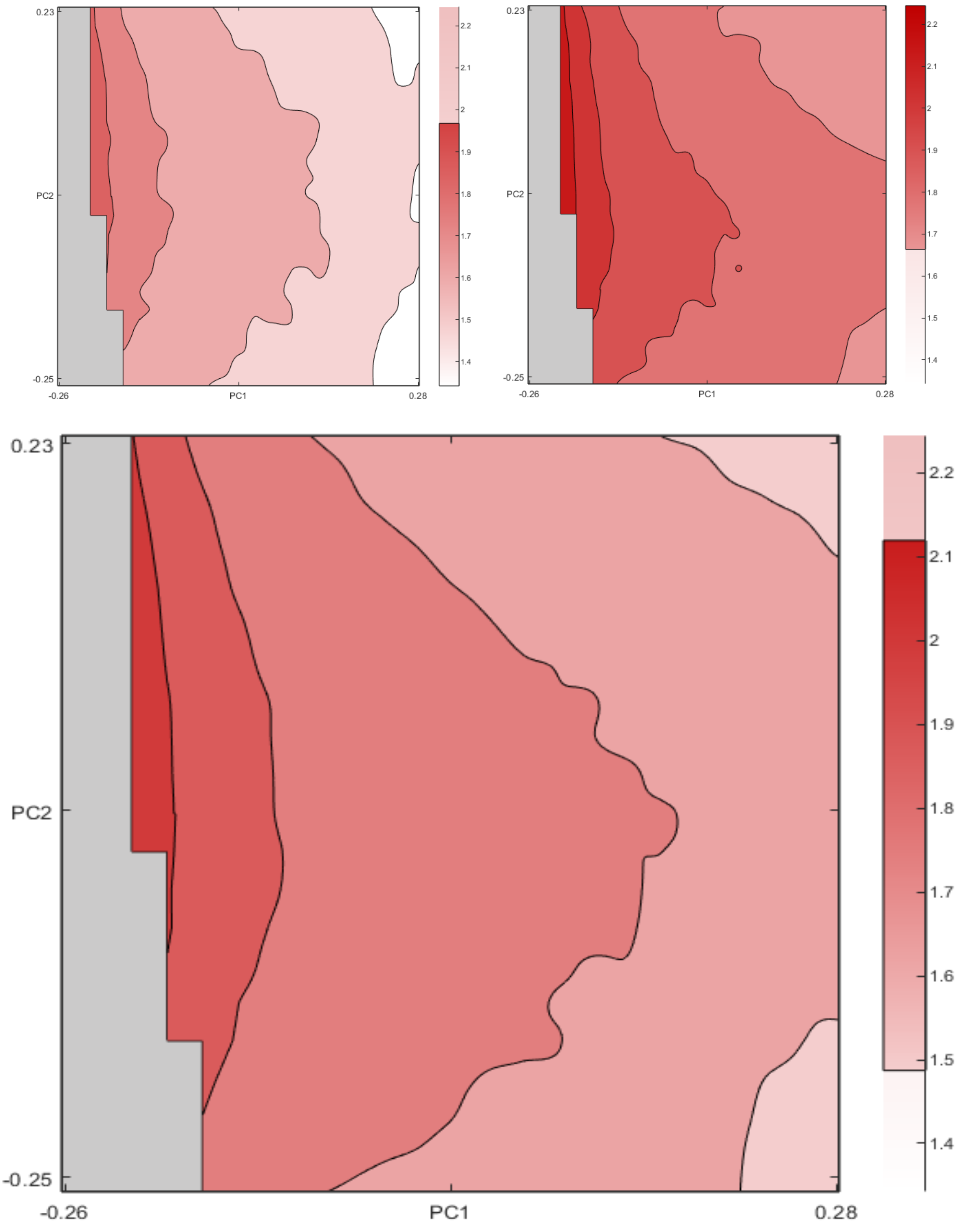

Fig S6: Rotational Efficiency (RE) surface plots. Top left: 5th percentile result of 1000 randomised inputs. Top right: 95th percentile result of 1000 randomised inputs. Bottom: mean result of 1000 randomised inputs.

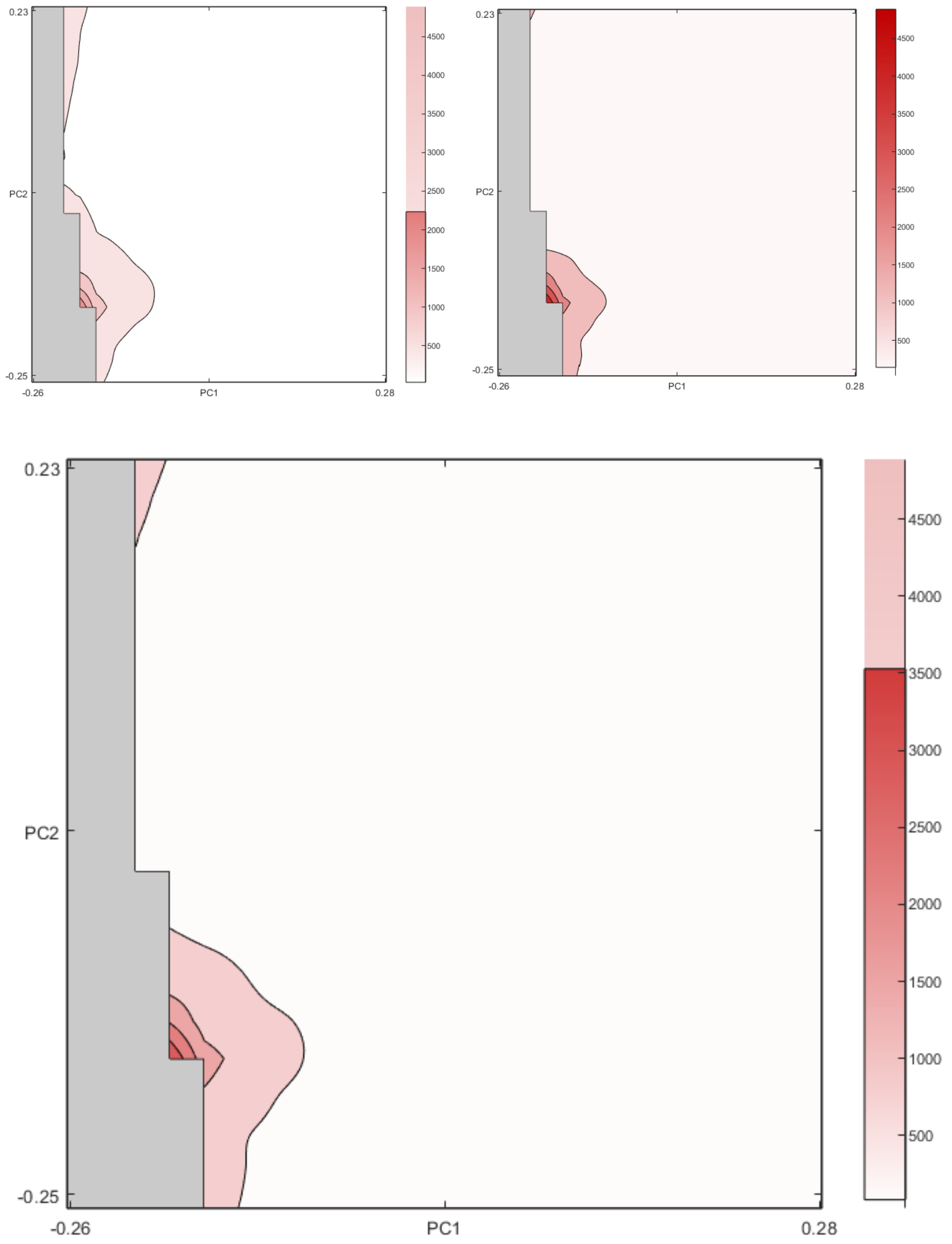

Fig S7: Von Mises Stress (VMS) surface plots. Top left: 5th percentile result of 1000 randomised inputs. Top right: 95th percentile result of 1000 randomised inputs. Bottom: mean result of 1000 randomised inputs.

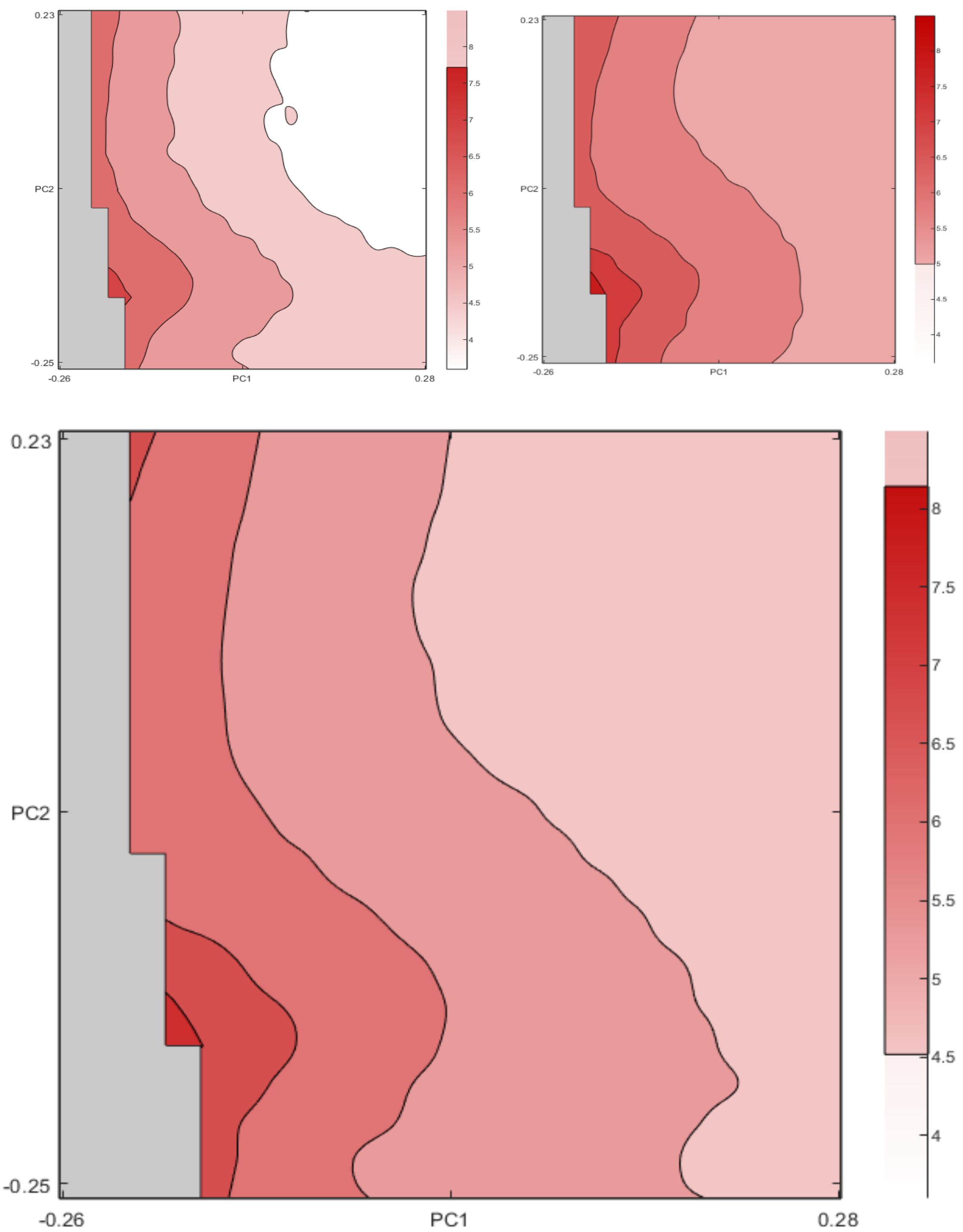

Fig S8: Log scaled Von Mises Stress (log VMS) surface plots. Top left: 5th percentile result of 1000 randomised inputs. Top right: 95th percentile result of 1000 randomised inputs. Bottom: mean result of 1000 randomised inputs.

Phylogeny

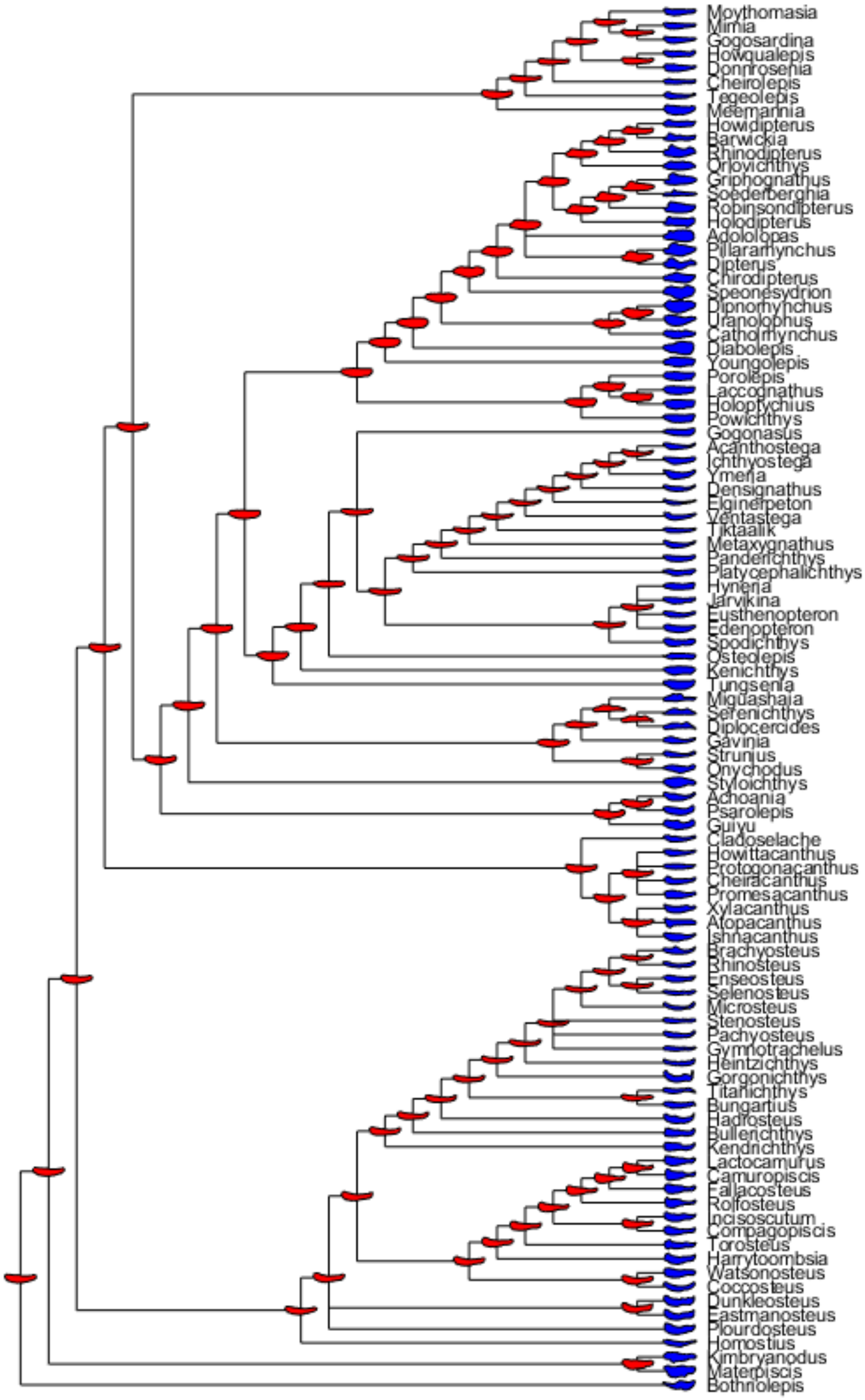

Fig S9: Phylogeny with taxon jaw shapes and ancestral jaw shapes superimposed. Blue shapes = empirical jaw shapes of sample taxa, red shapes = reconstructed jaw shapes output from ancestral state reconstruction.

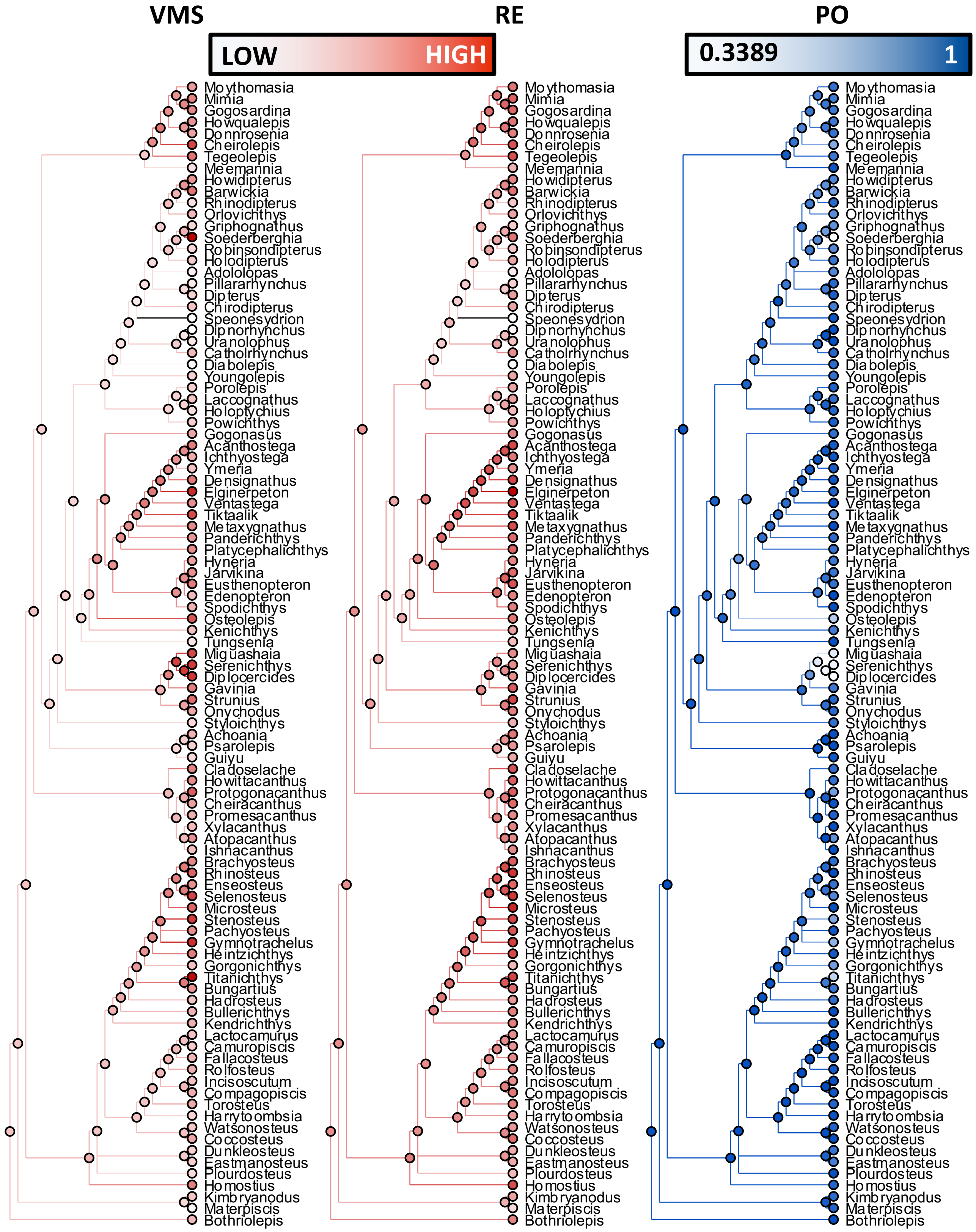

Fig S10: Phylogeny with extrapolated performance values (shades of red) and optimality values (shades of blue) superimposed. Coloured circles represent values of individual nodes (both empirical taxa and inferred ancestral states). VMS = Von Mises Stress; RE = Rotational Efficiency; PO = Pareto Optimality.

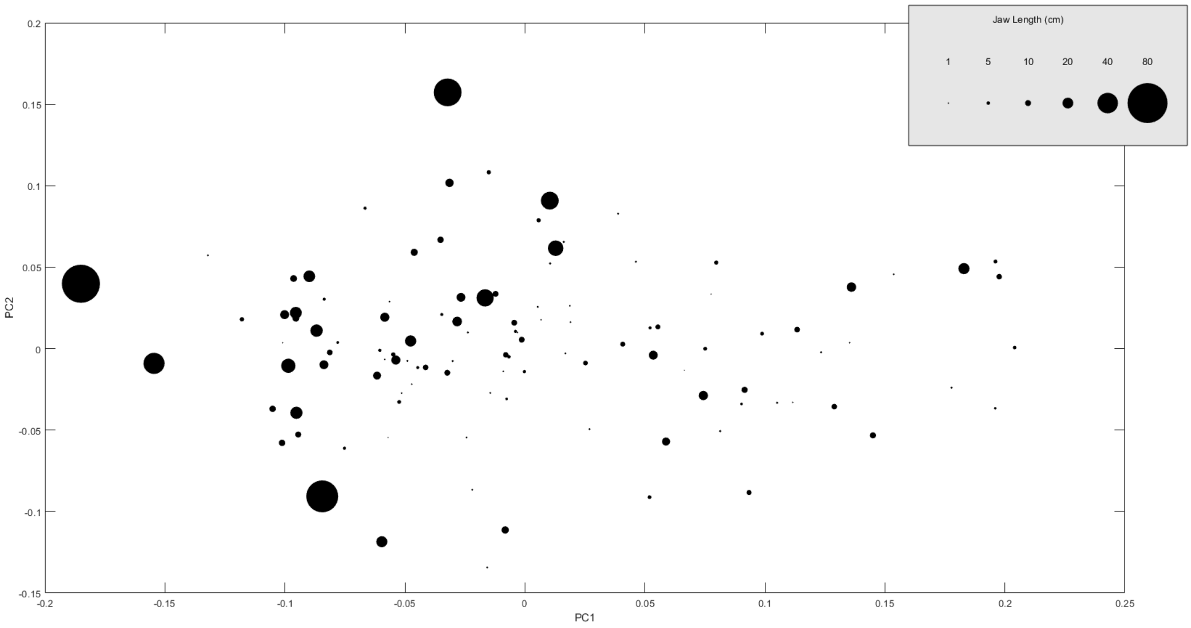

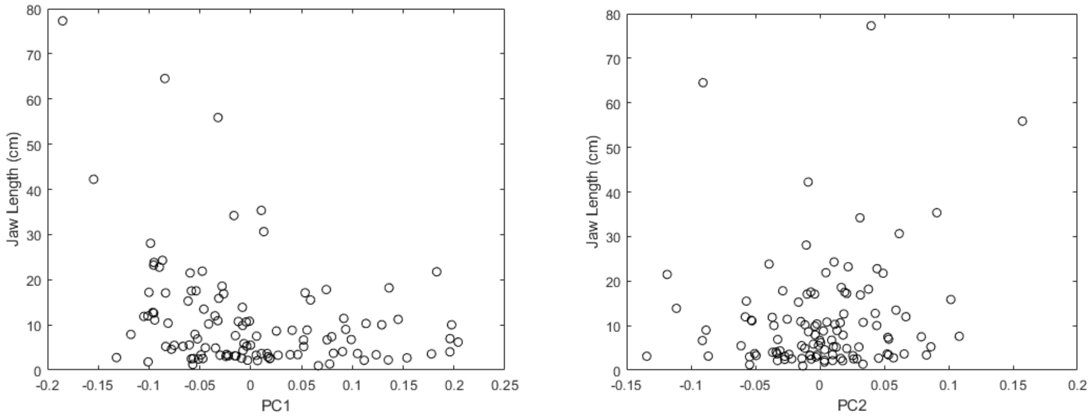

Fig

S11: Relationship between jaw length and shape. Longer Jaws appear to be biased towards lower PC1 values, but all PC2 values. Shorter jaws are less restricted in shape than longer jaws. A PGLS shows a significant relationship between jaw length and PC1 (p = 0.04129, R-squared = 0.03485), and no significant relationship between length and PC2 (p = 0.4846, R-squared = 0.0056).

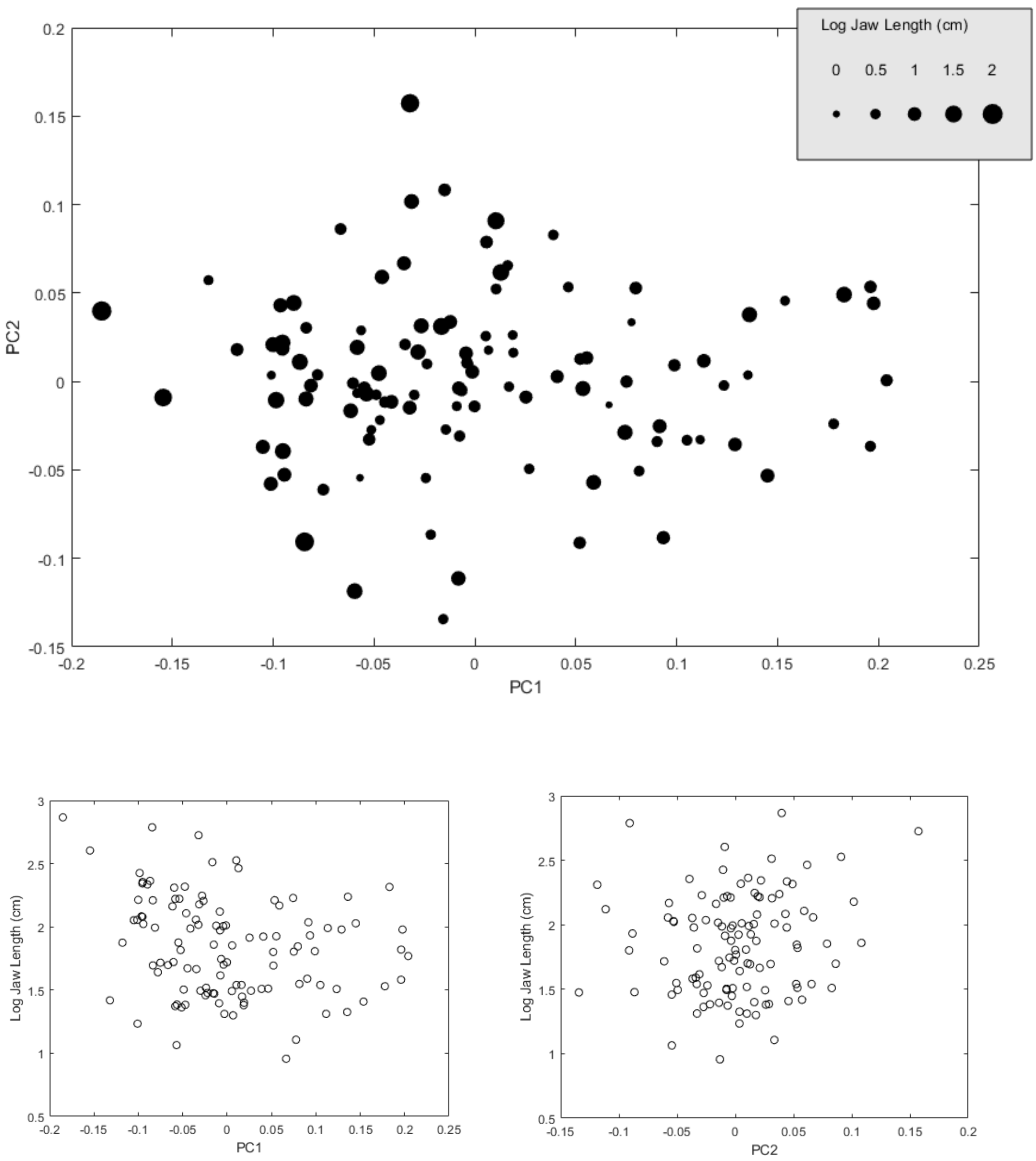

Fig S12: Relationship between log jaw length and shape. A PGLS shows a significant relationship between jaw length and PC1 ($p = 0.00462$, R-squared = 0.0627), and no significant relationship between length and PC2 ($p = 0.3191$, R-squared = 0.00954).

Data S1: Data sources, polynomial fit of performance surfaces, and disparity metrics

Data S1A: Sources of mandible morphology data

Index Number	Genus	First Appearance	Last Appearance	Clade	Image Type	Image Source
1	*Acanthostega*	Frasnian	Famennian	Sarcopterygii	Reconstruction	(*83*)
2	*Achoania*	Lochkovian	Lochkovian	Sarcopterygii	Specimen Photograph	(*84*)
3	*Adololopas*	Frasnian	Frasnian	Sarcopterygii	Specimen Photograph	(*85*)
4	*Angarichthys*	Eifelian	Givetian	Placodermi	Specimen Photograph	(*86*)
5	*Arquatichthys*	Pragian	Pragian	Sarcopterygii	Specimen Drawing	(*87*)
6	*Atopacanthus*	Givetian	Givetian	Chondrichthyes	Specimen Photograph	(*88*)
7	*Barwickia*	Givetian	Frasnian	Sarcopterygii	Reconstruction	(*89*)
8	*Bothriolepis*	Frasnian	Famennian	Placodermi	µCT Scan	(*90*)
9	*Brachydeirus*	Frasnian	Frasnian	Placodermi	Specimen Photograph	NHM: 53180
10	*Brachyosteus*	Frasnian	Frasnian	Placodermi	Reconstruction	(*91*)
11	*Brontichthys*	Famennian	Famennian	Placodermi	Specimen Photograph	CMNH: 7575
12	*Bullerichthys*	Frasnian	Frasnian	Placodermi	Specimen Photograph	WAM: 869703
13	*Bungartius*	Famennian	Famennian	Placodermi	Specimen Drawing	(*92*)
14	*Camuropiscis*	Frasnian	Frasnian	Placodermi	Specimen Photograph	(*5*)
15	*Cathlorhynchus*	Lochkovian	Eifelian	Sarcopterygii	Specimen Photograph	(*93*)
16	*Cavanosteus*	Emsian	Emsian	Placodermi	Specimen Photograph	ANU: V77
17	*Cheiracanthus*	Eifelian	Givetian	Chondrichthyes	Specimen Photograph	UCMZ: 1132
18	*Cheirolepis*	Emsian	Frasnian	Actinopterygii	Specimen Photograph	MNB: 5112
19	*Chirodipterus*	Frasnian	Frasnian	Sarcopterygii	Specimen Photograph	MNB: 12875a
20	*Cladoselache*	Famennian	Famennian	Chondrichthyes	Specimen Drawing	(*94*)
21	*Coccosteus*	Eifelian	Givetian	Placodermi	Specimen Photograph	(*95*)
22	*Compagopiscis*	Frasnian	Frasnian	Placodermi	µCT Scan	(*90*)
23	*Copanognathus*	Frasnian	Frasnian	Placodermi	Specimen Drawing	(*96*)
24	*Denisonodus*	Frasnian	Frasnian	Placodermi	Specimen Photograph	(*97*)
25	*Densignathus*	Famennian	Famennian	Sarcopterygii	Reconstruction	(*98*)
26	*Diabolepis*	Lochkovian	Lochkovian	Sarcopterygii	Specimen Photograph	(*99*)
27	*Diplocercides*	Givetian	Frasnian	Sarcopterygii	Reconstruction	(*100*)
28	*Diplognathus*	Famennian	Famennian	Placodermi	Reconstruction	(*91*)
29	*Dipnorhynchus*	Emsian	Emsian	Sarcopterygii	Specimen Photograph	NHM: 46773

30	*Dipterus*	Famennian	Famennian	Sarcopterygii	Specimen Photograph	NHM: P34552
31	*Donnrosenia*	Givetian	Givetian	Actinopterygii	Specimen Photograph	(*72*)
32	*Dunkleosteus*	Famennian	Famennian	Placodermi	Specimen Photograph	(*5*)
33	*Eastmanosteus*	Givetian	Famennian	Placodermi	Specimen Photograph	(*5*)
34	*Edenopteron*	Famennian	Famennian	Sarcopterygii	Reconstruction	(*101*)
35	*Elginerpeton*	Frasnian	Frasnian	Sarcopterygii	Reconstruction	(*83*)
36	*Enseosteus*	Frasnian	Frasnian	Placodermi	Specimen Photograph	MNB: 18318
37	*Erromenosteus*	Frasnian	Frasnian	Placodermi	Specimen Photograph	MNB: 139
38	*Eusthenopteron*	Givetian	Famennian	Sarcopterygii	Reconstruction	(*102*)
39	*Fallacosteus*	Frasnian	Frasnian	Placodermi	Specimen Photograph	(*5*)
40	*Gavinia*	Eifelian	Givetian	Sarcopterygii	Reconstruction	(*103*)
41	*Gogonasus*	Frasnian	Frasnian	Sarcopterygii	Specimen Photograph	(*104*)
42	*Gogosardina*	Frasnian	Frasnian	Actinopterygii	Specimen Photograph	(*105*)
43	*Gorgonichthys*	Famennian	Famennian	Placodermi	Specimen Photograph	(*5*)
44	*Griphognathus*	Frasnian	Frasnian	Sarcopterygii	Specimen Photograph	ANU: 21186
45	*Guiyu*	Silurian	Silurian	Sarcopterygii	Specimen Photograph	(*70*)
46	*Gymnotrachelus*	Famennian	Famennian	Placodermi	Specimen Photograph	CMNH: 8051
47	*Hadrosteus*	Frasnian	Frasnian	Placodermi	Reconstruction	(*91*)
48	*Harrytoombsia*	Frasnian	Frasnian	Placodermi	Specimen Photograph	WAM: 704254
49	*Heintzichthys*	Frasnian	Famennian	Placodermi	Specimen Photograph	(*106*)
50	*Holodipterus*	Frasnian	Frasnian	Sarcopterygii	Reconstruction	(*100*)
51	*Holodus*	Frasnian	Frasnian	Sarcopterygii	Specimen Drawing	(*107*)
52	*Holoptychius*	Givetian	Famennian	Sarcopterygii	Specimen Photograph	(*108*)
53	*Homostius*	Emsian	Givetian	Placodermi	Specimen Photograph	(*109*)
54	*Howidipterus*	Frasnian	Frasnian	Sarcopterygii	Reconstruction	(*89*)
55	*Howittacanthus*	Givetian	Givetian	Chondrichthyes	Specimen Drawing	(*110*)
56	*Howqualepis*	Givetian	Givetian	Actinopterygii	Specimen Drawing	(*111*)
57	*Hussakofia*	Famennian	Famennian	Placodermi	Specimen Photograph	CMNH: 8082
58	*Hyneria*	Famennian	Famennian	Sarcopterygii	Specimen Drawing	(*112*)
59	*Ichthyostega*	Famennian	Famennian	Sarcopterygii	Reconstruction	(*83*)
60	*Incisoscutum*	Frasnian	Frasnian	Placodermi	Specimen Photograph	(*106*)

61	*Ishnacanthus*	Silurian	Eifelian	Chondrichthyes	Specimen Photograph	(*113*)
62	*Jarvikina*	Givetian	Famennian	Sarcopterygii	Specimen Photograph	(*114*)
63	*Kendrichthys*	Frasnian	Frasnian	Placodermi	Specimen Photograph	NHM: P51143
64	*Kenichthys*	Emsian	Emsian	Sarcopterygii	Reconstruction	(*84*)
65	*Kimbryanodus*	Frasnian	Frasnian	Placodermi	Specimen Photograph	(*115*)
66	*Laccognathus*	Givetian	Frasnian	Sarcopterygii	Specimen Photograph	(*116*)
67	*Lactocamurus*	Frasnian	Frasnian	Placodermi	Specimen Photograph	(*5*)
68	*Latvius*	Givetian	Frasnian	Sarcopterygii	Reconstruction	MNB: f551
69	*Leptosteus*	Frasnian	Frasnian	Placodermi	Specimen Photograph	MNB: 12709
70	*Malerosteus*	Frasnian	Frasnian	Placodermi	Specimen Photograph	(*117*)
71	*Materpiscis*	Frasnian	Frasnian	Placodermi	Specimen Drawing	(*118*)
72	*Meemannia*	Lochkovian	Lochkovian	Actinopterygii	Specimen Drawing	(*119*)
73	*Megamastax*	Silurian	Silurian	Sarcopterygii	Specimen Photograph	(*120*)
74	*Metaxygnathus*	Famennian	Famennian	Sarcopterygii	Reconstruction	(*83*)
75	*Microsteus*	Frasnian	Frasnian	Placodermi	Specimen Photograph	MNB: 18324
76	*Miguashaia*	Givetian	Frasnian	Sarcopterygii	Reconstruction	(*121*)
77	*Mimia*	Frasnian	Frasnian	Actinopterygii	Reconstruction	(*84*)
78	*Moythomasia*	Givetian	Famennian	Actinopterygii	Specimen Drawing	NHM: P53221
79	*Mylostoma*	Famennian	Famennian	Placodermi	Specimen Photograph	CMNH: 7706
80	*Nesides*	Frasnian	Frasnian	Sarcopterygii	Reconstruction	(*103*)
81	*Onychodus*	Emsian	Famennian	Sarcopterygii	Reconstruction	(*103*)
82	*Orlovichthys*	Famennian	Famennian	Sarcopterygii	Specimen Drawing	(*122*)
83	*Osteolepis*	Eifelian	Givetian	Sarcopterygii	Specimen Photograph	UCMZ: GN769
84	*Oxyosteus*	Frasnian	Frasnian	Placodermi	Specimen Photograph	MNB: 296
85	*Pachyosteus*	Frasnian	Famennian	Placodermi	Specimen Photograph	MNB: 389
86	*Paledaphus*	Frasnian	Famennian	Sarcopterygii	Specimen Photograph	AMNH: 6560
87	*Panderichthys*	Givetian	Frasnian	Sarcopterygii	Specimen Drawing	(*83*)
88	*Pholidosteus*	Frasnian	Frasnian	Placodermi	Specimen Photograph	MNB: 12797
89	*Pillararhynchus*	Frasnian	Frasnian	Sarcopterygii	Specimen Photograph	(*123*)
90	*Platycephalichthys*	Frasnian	Frasnian	Sarcopterygii	Reconstruction	(*7*)
91	*Plourdosteus*	Givetian	Frasnian	Placodermi	Specimen Photograph	(*124*)

92	*Porolepis*	Lochkovian	Pragian	Sarcopterygii	Specimen Photograph	(*125*)
93	*Powichthys*	Lochkovian	Lochkovian	Sarcopterygii	Reconstruction	(*126*)
94	*Promesacanthus*	Lochkovian	Lochkovian	Chondrichthyes	Specimen Drawing	(*127*)
95	*Protogonacanthus*	Frasnian	Famennian	Chondrichthyes	Reconstruction	(*128*)
96	*Psarolepis*	Silurian	Lochkovian	Sarcopterygii	Specimen Photograph	(*84*)
97	*Ptyctodus*	Eifelian	Famennian	Placodermi	Specimen Photograph	(*96*)
98	*Rhinodipterus*	Frasnian	Famennian	Sarcopterygii	Specimen Photograph	(*123*)
99	*Rhinosteus*	Frasnian	Frasnian	Placodermi	Reconstruction	(*91*)
100	*Robinsondipterus*	Frasnian	Frasnian	Sarcopterygii	Specimen Photograph	WAM: 011003
101	*Rolfosteus*	Frasnian	Frasnian	Placodermi	Specimen Photograph	NHM: P50971
102	*Selenosteus*	Frasnian	Famennian	Placodermi	Specimen Photograph	CMNH: 8086
103	*Serenichthys*	Famennian	Famennian	Sarcopterygii	Reconstruction	(*129*)
104	*Soederberghia*	Frasnian	Famennian	Sarcopterygii	Reconstruction	(*100*)
105	*Speonesydrion*	Pragian	Pragian	Sarcopterygii	Specimen Photograph	ANU: 35647
106	*Spodichthys*	Frasnian	Frasnian	Sarcopterygii	µCT Scan	(*130*)
107	*Stenognathus*	Frasnian	Frasnian	Placodermi	Specimen Drawing	(*96*)
108	*Stenosteus*	Frasnian	Famennian	Placodermi	Specimen Photograph	CMNH: 8044
109	*Strunius*	Givetian	Famennian	Sarcopterygii	Specimen Photograph	MNB: 161b
110	*Styloichthys*	Lochkovian	Lochkovian	Sarcopterygii	Specimen Photograph	(*84*)
111	*Tegeolepis*	Frasnian	Famennian	Actinopterygii	Specimen Photograph	NHM: P45312
112	*Tiktaalik*	Frasnian	Frasnian	Sarcopterygii	Specimen Photograph	(*131*)
113	*Titanichthys*	Famennian	Famennian	Placodermi	Specimen Photograph	(*132*)
114	*Torosteus*	Frasnian	Frasnian	Placodermi	Specimen Photograph	WAM: 8863
115	*Tungsenia*	Pragian	Pragian	Sarcopterygii	Reconstruction	(*133*)
116	*Uranolophus*	Pragian	Eifelian	Sarcopterygii	Specimen Photograph	FMNH: PF3874
117	*Ventastega*	Famennian	Famennian	Sarcopterygii	Specimen Drawing	(*83*)
118	*Watsonosteus*	Eifelian	Givetian	Placodermi	Specimen Drawing	(*134*)
119	*Xylacanthus*	Silurian	Pragian	Chondrichthyes	Specimen Photograph	(*135*)
120	*Ymeria*	Frasnian	Famennian	Sarcopterygii	Reconstruction	(*136*)
121	*Youngolepis*	Lochkovian	Pragian	Sarcopterygii	Reconstruction	(*84*)

Abbreviation	Institution
NHM	Natural History Museum, London, UK

MNB	Museum für Naturkunde, Berlin, Germany
CMNH	Cleveland Museum of Natural History, Cleveland, OH, USA
WAM	Western Australian Museum, Perth, Australia
ANU	Australian National University, Canberra, Australia
UCMZ	University of Cambridge Museum of Zoology, Cambridge, UK
FMNH	The Field Museum, Chicago, IL, USA
AMNH	American Museum of natural History, New York, NY, USA

Data S1B: Second order polynomial fit to Rotational Efficiency (RE) and Von Mises Stress (VMS) surface

Equation:

	RE	RE 5% ci	RE 95% ci	VMS	VMS 5% ci	VMS 95% ci
p_C	1.805	1.8	1.81	255.5	225.6	285.5
p_x	-0.7324	-0.755	-0.7099	-1783	-1914	-1652
p_y	-0.04755	-0.06746	-0.02764	-734.9	-850.6	-619.3
p_{xx}	1.308	0.8923	1.185	5205	4356	6054
p_{xy}	-0.1133	-0.2447	0.01808	3364	2600	4127
p_{yy}	-1.837	-1.977	-1.697	1046	234	1858

Goodness of Fit:

	RE	VMS
**SSE**	0.3619	12210000
**R^2**	0.928	0.6669
**Adjusted R^2**	0.9272	0.663
**RMSE**	0.02929	170.1191

Data S1C: Spearman rank correlation test for trend over time (* = significant at p < 0.05)

	Sum of Variances	Mean Pairwise Distance	Mean Optimality
**rho**	0.6667	0.8810*	-0.9286*
**p**	0.0831	0.0072	0.0022

Data S1D: Pearson's linear rank test of disparity metrics against mean optimality (* = significant at p < 0.05)

	Sum of Variances	Mean Pairwise Distance
**r**	-0.5476	-0.7619*

**p**	0.171	0.0368

REFERENCES AND NOTES

1. P. C. J. Donoghue, J. N. Keating, Early vertebrate evolution. *Palaeontology* **57**, 879–893 (2014).

2. R. H. Denison, Feeding mechanisms of agnatha and early gnathostomes. *Am. Zool.* **1**, 177–181 (1961).

3. K. Dennis, R. S. Miles, New durophagous arthrodires from Gogo, Western-Australia. *Zool. J. Linnean Soc.* **69**, 43–85 (1980).

4. P. S. L. Anderson, M. W. Westneat, Feeding mechanics and bite force modelling of the skull of *Dunkleosteus terelli*, an ancient apex predator. *Biol. Lett.* **3**, 76–79 (2006).

5. E. Snively, P. S. L. Anderson, M. J. Ryan, Functional and ontogenetic implications of bite stress in arthrodire placoderms. *Kirtlandia* **57**, 53–60 (2009).

6. P. S. L. Anderson, M. Friedman, M. D. Brazeau, E. J. Rayfield, Initial radiation of jaws demonstrated stability despite faunal and environmental change. *Nature* **476**, 206–209 (2011).

7. P. S. L. Anderson, M. Friedman, M. Ruta, Late to the table: Diversification of tetrapod mandibular biomechanics lagged behind the evolution of terrestriality. *Integr. Comp. Biol.* **53**, 197–208 (2013).

8. F. P. Kuhl, C. R. Giardina, Elliptic fourier features of a closed contour. *Comput. Graph. Image Process.* **18**, 236–258 (1982).

9. G. R. McGhee Jr., Shell form in the biconvex articulate brachiopoda: A geometric analysis. *Paleobiology* **6**, 57–76 (1980).

10. G. R. McGhee, *The Geometry of Evolution: Adaptive Landscapes and Theoretical Morphospaces* (Cambridge Univ. Press, 2007), pp. 200.

11. J. J. Hill, M. N. Puttick, T. L. Stubbs, E. J. Rayfield, P. C. J. Donoghue, Evolution of jaw disparity in fishes. *Palaeontology* **61**, 847–854 (2018).

12. B. Figueirido, Z. J. Tseng, A. Martin-Serra, Skull shape evolution in durophagous carnivorans. *Evolution* **67**, 1975–1993 (2013).

13. M. N. Simon, R. Brandt, T. Kohlsdorf, S. J. Arnold, Bite performance surfaces of three ecologically divergent Iguanidae lizards: Relationships with lower jaw bones. *Biol. J. Linn. Soc.* **127**, 810–825 (2019).

14. P. C. Wainwright, M. E. Alfaro, D. I. Bolnick, C. D. Hulsey, Many-to-one mapping of form to function: A general principle in organismal design? *Integr. Comp. Biol.* **45**, 256–262 (2005).

15. P. D. Polly, C. T. Stayton, E. R. Dumont, S. E. Pierce, E. J. Rayfield, K. D. Angielczyk, Combining geometric morphometrics and finite element analysis with evolutionary modeling: Towards a synthesis. *J. Vertebr. Paleontol.* **36**, e1111225 (2016).

16. C. T. Stayton, Performance in three shell functions predicts the phenotypic distribution of hard-shelled turtles. *Evolution* **73**, 720–734 (2019).

17. C. T. Stayton, L. F. O’Connor, N. M. Nisivoccia, The influence of multiple functional demands on morphological diversification: A test on turtle shells. *Evolution* **72**, 1933–1949 (2018).

18. Z. J. Tseng, Testing adaptive hypotheses of convergence with functional landscapes: A case study of bone-cracking hypercarnivores. *PLOS ONE* **8**, e65305 (2013).

19. B. V. Dickson, S. E. Pierce, Functional performance of turtle humerus shape across an ecological adaptive landscape. *Evolution* **73**, 1265–1277 (2019).

20. S. J. Arnold, Morphology, performance and fitness. *Am. Zool.* **23**, 347–361 (1983).

21. S. J. Arnold, Performance surfaces and adaptive landscapes. *Integr. Comp. Biol.* **43**, 367–375 (2003).

22. C. H. Martin, P. C. Wainwright, Multiple fitness peaks on the adaptive landscape drive adaptive radiation in the wild. *Science* **339**, 208–211 (2013).

23. B. V. Dickson, J. A. Clack, T. R. Smithson, S. E. Pierce, Functional adaptive landscapes predict terrestrial capacity at the origin of limbs. *Nature* **589**, 242–245 (2021).

24. D. M. Raup, Computer as aid in describing form in gastropod shells. *Science* **138**, 150–152 (1962).

25. D. M. Raup, A. Michelson, Theoretical morphology of coiled shell. *Science* **147**, 1294–1295 (1965).

26. D. M. Raup, Geometry of coiling in gastropods. *Proc. Natl. Acad. Sci. U.S.A.* **47**, 602–609 (1961).

27. D. M. Raup, Geometric analysis of shell coiling in ammonoids. *J. Paleontol.* **41**, 43–65 (1967).

28. S. Van Wassenbergh, J. Brecko, P. Aerts, I. Stouten, G. Vanheusden, A. Camps, R. Van Damme, A. Herrel, Hydrodynamic constraints on prey-capture performance in forward-striking snakes. *J. R. Soc. Interface* **7**, 773–785 (2010).

29. S. W. Day, T. E. Higham, A. Y. Cheer, P. C. Wainwright, Spatial and temporal patterns of water flow generated by suction-feeding bluegill sunfishLepomis macrochirusresolved by particle image velocimetry. *J. Exp. Biol.* **208**, 2661–2671 (2005).

30. P. C. Wainwright, M. D. McGee, S. J. Longo, L. P. Hernandez, Origins, innovations, and diversification of suction feeding in vertebrates. *Integr. Comp. Biol.* **55**, 134–145 (2015).

31. S. Van Wassenbergh, S. W. Day, L. P. Hernandez, T. E. Higham, T. Skorczewski, Suction power output and the inertial cost of rotating the neurocranium to generate suction in fish. *J. Theor. Biol.* **372**, 159–167 (2015).

32. S. Van Wassenbergh, P. Aerts, D. Adriaens, A. Herrel, A dynamic model of mouth closing movements in clariid catfishes: The role of enlarged jaw adductors. *J. Theor. Biol.* **234**, 49–65 (2005).

33. C. P. Kenaley, Exploring feeding behaviour in deep-sea dragonfishes (Teleostei: Stomiidae): Jaw biomechanics and functional significance of a loosejaw. *Biol. J. Linn. Soc.* **106**, 224–240 (2012).

34. J. F. V. Vincent, The trade-off: A central concept for biomimetics. *Bioinspir. Biomim. Nanobiomaterials* **6**, 67–76 (2017).

35. D. C. Adams, A generalized K statistic for estimating phylogenetic signal from shape and other high-dimensional multivariate data. *Syst. Biol.* **63**, 685–697 (2014).

36. C. N. Ciampaglio, M. Kemp, D. W. McShea, Detecting changes in morphospace occupation patterns in the fossil record: Characterization and analysis of measures of disparity. *Paleobiology* **27**, 695–715 (2001).

37. M. M. Munoz, Y. Hu, P. S. L. Anderson, S. N. Patek, Strong biomechanical relationships bias the tempo and mode of morphological evolution. *eLife* **7**, (2018).

38. C. Gans, R. G. Northcutt, Neural crest and the origin of vertebrates: A new head. *Science* **220**, 268–273 (1983).

39. R. G. Northcutt, Evolution of the optic tectum in ray-finned fishes, in *Fish Neurobiology 2. Higher Brain Areas and Functions*, R. E. Davis, R. G. Northcutt, Eds. (The University of Michigan Press, 1983), vol. 2, pp. 1–42.

40. C. Gans, Stages in the origin of vertebrates: Analysis by means of scenarios. *Biol. Rev.* **64**, 221–268 (1989).

41. L. A. Ferry, E. M. Paig-Tran, A. C. Gibb, Suction, ram, and biting: Deviations and limitations to the capture of aquatic prey. *Integr. Comp. Biol.* **55**, 97–109 (2015).

42. S. J. Coatham, J. Vinther, E. J. Rayfield, C. Klug, Was the Devonian placoderm *Titanichthys* a suspension feeder? *R. Soc. Open Sci.* **7**, 200272 (2020).

43. A. M. Herbert, P. J. Motta, Biomechanics of the jaw of the durophagous bonnethead shark. *Zoology (Jena)* **129**, 54–58 (2018).

44. J. Mallatt, Ventilation and the origin of jawed vertebrates: A new mouth. *Zool. J. Linnean Soc.* **117**, 329–404 (1996).

45. T. Miyashita, Fishing for jaws in early vertebrate evolution: A new hypothesis of mandibular confinement. *Biol. Rev.* **91**, 611–657 (2016).

46. P. S. L. Anderson, Cranial muscle homology across modern gnathostomes. *Biol. J. Linn. Soc.* **94**, 195–216 (2008).

47. M. Hughes, S. Gerber, M. A. Wills, Clades reach highest morphological disparity early in their evolution. *Proc. Natl. Acad. Sci. U.S.A.* **110**, 13875–13879 (2013).

48. L. J. Harmon, J. B. Losos, T. J. Davies, R. G. Gillespie, J. L. Gittleman, W. Bryan Jennings, K. H. Kozak, M. A. McPeek, F. Moreno-Roark, T. J. Near, A. Purvis, R. E. Ricklefs, D. Schluter, J. A. Schulte Ii, O. Seehausen, B. L. Sidlauskas, O. Torres-Carvajal, J. T. Weir, A. O. Mooers, Early bursts of body size and shape evolution are rare in comparative data. *Evolution* **64**, 2385–2396 (2010).

49. J. Marce-Nogue, T. A. Puschel, A. Daasch, T. M. Kaiser, Broad-scale morpho-functional traits of the mandible suggest no hard food adaptation in the hominin lineage. *Sci. Rep.* **10**, 6793 (2020).

50. N. M. Morales-Garcia, T. D. Burgess, J. J. Hill, P. G. Gill, E. J. Rayfield, The use of extruded finite-element models as a novel alternative to tomography-based models: A case study using early mammal jaws. *J. R. Soc. Interface* **16**, 20190674 (2019).

51. M. Ruta, J. Krieger, K. D. Angielczyk, M. A. Wills, The evolution of the tetrapod humerus: Morphometrics, disparity, and evolutionary rates. *Earth Environ. Sci. Trans. R. Soc. Edinb.* **109**, 351–369 (2018).

52. J. M. Carlo, M. S. Barbeitos, H. R. Lasker, Quantifying complex shapes: Elliptical fourier analysis of octocoral sclerites. *Biol. Bull.* **220**, 224–237 (2011).

53. F. Márquez, J. Robledo, G. E. Peñaloza, S. Van der Molen, Use of different geometric morphometrics tools for the discrimination of phenotypic stocks of the striped clam *Ameghinomya antiqua* (Veneridae) in north Patagonia, Argentina. . *Fish. Res.* **101**, 127–131 (2010).

54. T. J. Challands, T. R. Smithson, J. A. Clack, C. E. Bennett, J. E. A. Marshall, S. M. Wallace-Johnson, H. Hill, A lungfish survivor of the end-Devonian extinction and an Early Carboniferous dipnoan radiation. *J. Syst. Palaeontol.* **17**, 1825–1846 (2019).

55. L. Frey, M. Coates, M. Ginter, V. Hairapetian, M. Rucklin, I. Jerjen, C. Klug, The early elasmobranch *Phoebodus*: Phylogenetic relationships, ecomorphology and a new time-scale for shark evolution. *Proc. Biol. Sci.* **286**, 20191336 (2019).

56. A. M. Clement, B. King, S. Giles, B. Choo, P. E. Ahlberg, G. C. Young, J. A. Long, Neurocranial anatomy of an enigmatic Early Devonian fish sheds light on early osteichthyan evolution. *eLife* **7**, e34349 (2018).

57. J. A. Clack, T. J. Challands, T. R. Smithson, K. Z. Smithson, . Newly recognized Famennian lungfishes from East Greenland reveal tooth plate diversity and blur the Devonian–Carboniferous boundary. *Pap. Palaeontol.* **5**, 261–279 (2019).

58. B. Choo, J. Lu, S. Giles, K. Trinajstic, J. A. Long, A. Smith, A new actinopterygian from the Late Devonian Gogo Formation, Western Australia. . *Pap. Palaeontol.* **5**, 343–363 (2019).

59. J. D. Pardo, M. Szostakiwskyj, P. E. Ahlberg, J. S. Anderson, Hidden morphological diversity among early tetrapods. *Nature* **546**, 642–645 (2017).

60. N. Takezaki, H. Nishihara, Support for lungfish as the closest relative of tetrapods by using slowly evolving ray-finned fish as the outgroup. *Genome Biol. Evol.* **9**, 93–101 (2017).

61. A. Kemp, L. Cavin, G. Guinot, Evolutionary history of lungfishes with a new phylogeny of post-Devonian genera. *Palaeogeogr. Palaeoclimatol. Palaeoecol.* **471**, 209–219 (2017).

62. B. King, T. Qiao, M. S. Y. Lee, M. Zhu, J. A. Long, Bayesian morphological clock methods resurrect placoderm monophyly and reveal rapid early evolution in jawed vertebrates. *Syst. Biol.* **66**, 499–516 (2017).

63. M. I. Coates, J. A. Finarelli, I. J. Sansom, P. S. Andreev, K. E. Criswell, K. Tietjen, M. L. Rivers, P. J. La Riviere, An early chondrichthyan and the evolutionary assembly of a shark body plan. *Proc. Biol. Sci.* **285**, 20172418 (2018).

64. M. Zhu, P. E. Ahlberg, Z. Pan, Y. Zhu, T. Qiao, W. Zhao, L. Jia, J. Lu, A Silurian maxillate placoderm illuminates jaw evolution. *Science* **354**, 334–336 (2016).

65. S. Giles, M. Friedman, M. D. Brazeau, Osteichthyan-like cranial conditions in an Early Devonian stem gnathostome. *Nature* **520**, 82–85 (2015).

66. M. D. Brazeau, M. Friedman, The origin and early phylogenetic history of jawed vertebrates. *Nature* **520**, 490–497 (2015).

67. V. Dupret, S. Sanchez, D. Goujet, P. Tafforeau, P. E. Ahlberg, A primitive placoderm sheds light on the origin of the jawed vertebrate face. *Nature* **507**, 500–503 (2014).

68. B. Choo, Revision of the actinopterygian genus *Mimipiscis* (=*Mimia*) from the Upper Devonian Gogo Formation of Western Australia and the interrelationships of the early Actinopterygii. *Earth Environ. Sci. Trans. R. Soc. Edinb.* **102**, 77–104 (2012).

69. K. Trinajstic, K. Dennis-Bryan, Phenotypic plasticity, polymorphism and phylogeny within placoderms. *Acta Zool.* **90**, 83–102 (2009).

70. M. Zhu, W. Zhao, L. Jia, J. Lu, T. Qiao, Q. Qu, The oldest articulated osteichthyan reveals mosaic gnathostome characters. *Nature* **458**, 469–474 (2009).

71. M. D. Brazeau, The braincase and jaws of a Devonian "acanthodian" and modern gnathostome origins. *Nature* **457**, 305–308 (2009).

72. J. A. Long, B. Choo, G. C. Young, A new basal actinopterygian fish from the Middle Devonian Aztec Siltstone of Antarctica. *Antarct. Sci.* **20**, 393–412 (2008).

73. D. W. Bapst, paleotree: An R package for paleontological and phylogenetic analyses of evolution. *Methods Ecol. Evol.* **3**, 803–807 (2012).

74. D. C. Adams, E. Otárola-Castillo, E. Paradis, geomorph: An R package for the collection and analysis of geometric morphometric shape data. *Methods Ecol. Evol.* **4**, 393–399 (2013).

75. P. D. Polly, Functional tradeoffs carry phenotypes across the valley of the shadow of death. *Integr. Comp. Biol.* **60**, 1268–1282 (2020).

76. Z. J. Tseng, J. L. McNitt-Gray, H. Flashner, X. Wang, R. Enciso, Model sensitivity and use of the comparative finite element method in mammalian jaw mechanics: Mandible performance in the gray wolf. *PLOS ONE* **6**, e19171 (2011).

77. D. Adriaens, A. Lakhtakia, R. J. Martín-Palma, M. Knez, paper presented at the Bioinspiration, Biomimetics, and Bioreplication IX, 2019.

78. O. Shoval, H. Sheftel, G. Shinar, Y. Hart, O. Ramote, A. Mayo, E. Dekel, K. Kavanagh, U. Alon, Evolutionary trade-offs, pareto optimality, and the geometry of phenotype space. *Science* **336**, 1157–1160 (2012).

79. A. Tendler, A. Mayo, U. Alon, Evolutionary tradeoffs, Pareto optimality and the morphology of ammonite shells. *BMC Syst. Biol.* **9**, 12 (2015).

80. D. E. Goldberg, *Genetic Algorithms in Search, Optimization and Machine Learning* (Addison-Wesley Longman Publishing Co. Inc., 1989).

81. C. M. Fonseca, P. J. Fleming, Multiobjective optimization and multiple constraint handling with evolutionary algorithms–part I: A unified formulation. *IEEE Trans. Syst. Man Cybern. 1 Syst. Humans* **28**, 26–37 (1998).

82. I. Alberto, C. Azcarate, F. Mallor, P. M. Mateo, Multiobjective evolutionary algorithms. Pareto rankings. *Monografias del Semin. Matem. García de Galdeano* **27**, 27–35 (2003).

83. P. E. Ahlberg, J. A. Clack, Lower jaws, lower tetrapods–A review based on the Devonian genus *Acanthostega*. *Trans. R. Soc. Edinb. Earth Sci.* **89**, 11–46 (1998).

84. M. Zhu, X. Yu, Lower jaw character transitions among major sarcopterygian groups–A survey based on new materials from Yunnan, China, in *Recent Advances in the Origin and Early Radiation of Vertebrates*, G. Arratia, M. V. H. Wilson, R. Cloutier, Eds. (Pfeil, 2004), pp. 271–286.

85. K. S. W. Campbell, R. E. Barwick, A new tooth-plated dipnoan from the Upper Devonian Gogo Formation and its relationships. *Mem. Queensl. Mus.* **42**, 403–437 (1998).

86. A. Heintz, Fischreste aus dem Unterperm Norwegens. *Norsk Geol. Tidsskr.* **14**, 176–194 (1934).

87. J. Lu, M. Zhu, An Early Devonian (Pragian) sarcopterygian from Zhaotong, Yunnan, China. *Vertebrata PalAsiatica* **46**, 161 (2008).

88. H. Jessen, Weitere Fischreste aus dem Oberen Plattenkalk der Bergisch-Gladbach-Paffrather Mulde (Oberdevon, Rheinisches Schiefergebirge). *Palaeontographica Abt. A* **143**, 159–187 (1973).

89. J. A. Long, Cranial anatomy of two new late Devonian lungfishes (Pisces: Dipnoi) from Mount Howitt, Victoria. *Rec. Aust. Mus.* **44**, 299–318 (1992).

90. M. Rücklin, P. C. Donoghue, Z. Johanson, K. Trinajstic, F. Marone, M. Stampanoni, Development of teeth and jaws in the earliest jawed vertebrates. *Nature* **491**, 748–751 (2012).

91. R. K. Carr, Placoderm diversity and evolution. *Bull. Mus. Natl. Hist. Nat. Paris* **17**, 85–125 (1995).

92. D. H. Dunkle, A new genus and species of arthrodiran fish from the Upper Devonian Cleveland Shale. *Sci. Publ. Cleve. Mus. Nat. Hist.* **8**, 103–117 (1947).

93. K. S. W. Campbell, R. E. Barwick, T. J. Senden, Evolution of dipnoans (lungfish) in the Early Devonian of southeastern Australia. *Alcheringa* **33**, 59–78 (2009).

94. J. E. Harris, The neurocranium and jaws of *Cladoselache*. *Sci. Publ. Cleve. Mus. Nat. Hist.* **8**, 7–12 (1938).

95. A. Heintz, Revision of the structure of *Coccosteus decipiens* Ag. *Norsk Geol. Tidsskr.* **12**, 291–313 (1931).

96. L. Hussakof, W. L. Bryant, Catalogue of the fossil fishes in the museum of the Buffalo Society of Natural Sciences. *Buffalo Soc. Nat. Hist. Sci. Bull.* **12**, 1–346 (1919).

97. H. G. Johnson, D. K. Elliott, A new ptyctodont (Placodermi) from the Upper Devonian Martin Formation of northern Arizona, and an analysis of ptyctodont phylogeny. *J. Paleontol.* **70**, 994–1003 (1996).

98. E. B. Daeschler, Early tetrapod jaws from the Late Devonian of Pennsylvania, USA. . *J. Paleontol.* **74**, 301–308 (2000).

99. M. M. Smith, M.-M. Chang, The dentition of *Diabolepis speratus* Chang and Yu, with further consideration of its relationships and the primitive dipnoan dentition. *J. Vertebr. Paleontol.* **10**, 420–433 (1990).

100. M. Friedman, *Styloichthys* as the oldest coelacanth: Implications for early osteichthyan interrelationships. *J. Syst. Palaeontol.* **5**, 289–343 (2007).

101. B. Young, R. L. Dunstone, T. J. Senden, G. C. Young, A gigantic sarcopterygian (tetrapodomorph lobe-finned fish) from the upper Devonian of Gondwana (Eden, New South Wales, Australia). *PLOS ONE* **8**, e53871 (2013).

102. J. A. Clack, *Gaining Ground: The origin and Evolution of Tetrapods* (Indiana Univ. Press, 2012).

103. J. A. Long, A new genus of fossil coelacanth (Osteichthyes: Coelacanthiformes) from the Middle Devonian of southeastern Australia. *Rec. West. Aust. Mus.* **57**, 37–53 (1999).

104. J. A. Long, R. E. Barwick, K. S. W. Campbell, Osteology and functional morphology of the osteolepiform fish *Gogonasus andrewsae* Long, 1985, from the Upper Devonian Gogo Formation, Western Australia. *Rec. West. Aust. Mus.* **53**, 1-89 (1997).

105. B. Choo, J. A. Long, K. Trinajstic, A new genus and species of basal actinopterygian fish from the Upper Devonian Gogo Formation of Western Australia. *Acta Zool.* **90**, 194-210 (2009).

106. P. S. L. Anderson, Shape variation between arthrodire morphotypes indicates possible feeding niches. *J. Vertebr. Paleontol.* **28**, 961-969 (2008).

107. Z. Gorizdro-Kulczycka, Dwudyszne ryby dewońskie Gór Swiętokrzyskich. *Acta Geol. Pol.* **1**, 53-105 (1950).

108. J. P. Downs, *Holoptychius bergmanni* sp. nov.(Sarcopterygii, Porolepiformes) from the Upper Devonian of Nunavut, Canada, and a review of *Holoptychius* taxonomy. *Proc. Acad. Natl. Sci. Phila.* **162**, 47-59 (2013).

109. E. Mark-Kurik, The inferognathal in the Middle Devonian arthrodire *Homostius*. *Lethaia* **25**, 173-178 (1992).

110. J. A. Long, A new Late Devonian acanthodian fish from Mt. Howitt, Victoria, Australia, with remarks on acanthodian biogeography. *Proc. R. Soc. Victoria* **98**, 1-17 (1986).

111. J. A. Long, New palaeoniscoid fishes from the Late Devonian and Early Carboniferous of Victoria. *Mem. Assoc. Australas. Palaeontol.* **7**, 1-64 (1988).

112. E. B. Daeschler, J. P. Downs, New description and diagnosis of Hyneria lindae (Sarcopterygii, Tristichopteridae) from the Upper Devonian Catskill Formation in Pennsylvania, USA. *J. Vertebr. Paleontol.* **38**, e1448834 (2018).

113. E. I. White, The old red sandstone of brown clee Hill and the adjacent area II. Palaeontology, in *Bulletin of the British Museum (Natural History), Geology* [London: BM(NH), 1961], vol. 5, pp. 243–310.

114. E. I. Vorobyeva, Morphology and nature of evolution of crossopterygian fish. *Trudy Paleontologicheskogo Instituta* **163**, 1-239 (1977).

115. K. Trinajstic, J. A. Long, A new genus and species of Ptyctodont (Placodermi) from the Late Devonian Gneudna Formation, Western Australia, and an analysis of Ptyctodont phylogeny. *Geol. Mag.* **146**, 743-760 (2009).

116. E. I. Vorobyeva, A new species of *Laccognathus* (Porolepiform Crossopterygii) from the Devonian of Latvia. *Paleontol. Zhurnal* **40**, 312-322 (2006).

117. J. Kulczycki, Upper devonian fishes from the holy cross mountains. *Acta Palaeontol. Pol.* **2**, 285-380 (1957).

118. J. A. Long, K. Trinajstic, G. C. Young, T. Senden, Live birth in the Devonian period. *Nature* **453**, 650-652 (2008).

119. M. Zhu, W. Wang, X. Yu, *Meemania eos,* a basal sarcopterygian fish from the Lower Devonian of China–Expanded description and significance, in *Morphology, Phylogeny and palaeobIogeography of Fossil Fishes*, D. K. Elliott, J. G. Maisey, X. Yu, D. Miao, Eds. (Verlag Dr Friedrich Pfeil, 2010), pp. 199–214.

120. B. Choo, M. Zhu, W. Zhao, L. Jia, Y. Zhu, The largest Silurian vertebrate and its palaeoecological implications. *Sci. Rep.* **4**, 5242 (2015).

121. P. L. Forey, P. E. Ahlberg, E. Lukševičs, I. Zupinš, A new coelacanth from the Middle Devonian of Latvia. *J. Vertebr. Paleontol.* **20**, 243-252 (2000).

122. N. I. Krupina, R. R. Reisz, D. Scott, The skull and tooth system of *Orlovichthys limnatis*, a Late Devonian dipnoan from Russia. *Can. J. Earth Sci.* **38**, 1301-1311 (2001).

123. A. M. Clement, "The anatomy, evolution and interrelationships of Devonian Dipnoans, with insights from the extant Australian lungfish, *Neoceratodus forsteri*," thesis, Australian National University (2011).

124. T. Ørvig, Histologic studies of ostracoderms, placoderms and fossil elasmobranchs 3. Structure and growth of gnathalia of certain arthrodires. *Zool. Scr.* **9**, 141-159 (1980).

125. E. Jarvik, Middle and Upper Devonian porolepiformes from East Greenland with special reference to *Glyptolepis groenlandica* n.sp. and a discussion on the structure of the head in the porolepiformes. *Medd. Grønland* **187**, 1-307 (1972).

126. H. L. Jessen, Lower Devonian Porolepiformes from the Canadian Arctic with special reference to *Powichthys thorsteinssoni* Jessen. *Palaeontographica Abt. A* **167**, 180-214 (1980).

127. G. F. Hanke, *Promesacanthus eppleri* n. gen., n. sp., a mesacanthid (Acanthodii, Acanthodiformes) from the Lower Devonian of northern Canada. *Geodiversitas* **30**, 287-302 (2008).

128. R. S. Miles, The acanthodian fishes of the Devonian Plattenkalk of the Paffrath Trough in the Rhineland, with an appendix containing a classification of the Acanthodii and a revision of the genus *Homalacanthus*. *Arkiv Zool.* **18**, 147-194 (1966).

129. R. W. Gess, M. I. Coates, Fossil juvenile coelacanths from the Devonian of South Africa shed light on the order of character acquisition in actinistians. *Zool. J. Linnean Soc.* **175**, 360-383 (2015).

130. D. Snitting, A redescription of the anatomy of the Late DevonianSpodichthys buetleriJarvik, 1985 (Sarcopterygii, Tetrapodomorpha) from East Greenland. *J. Vertebr. Paleontol.* **28**, 637-655 (2008).

131. E. B. Daeschler, N. H. Shubin, F. A. Jenkins, A Devonian tetrapod-like fish and the evolution of the tetrapod body plan. *Nature* **440**, 757-763 (2006).

132. J. Boyle, M. J. Ryan, New information onTitanichthys(Placodermi, Arthrodira) from the Cleveland Shale (Upper Devonian) of Ohio, USA. *J. Paleontol.* **91**, 318-336 (2017).

133. J. Lu, M. Zhu, J. A. Long, W. Zhao, T. J. Senden, L. Jia, T. Qiao, The earliest known stem-tetrapod from the Lower Devonian of China. *Nat. Commun.* **3**, 1160 (2012).

134. R. S. Miles, T. S. Westoll, IX.—Two new genera of coccosteid arthrodira from the Middle Old Red Sandstone of Scotland, and their Stratigraphical distribution. *Trans. R. Soc. Edinburgh* **65**, 179-210 (1962).

135. T. Ørvig, Some new acanthodian material from the Lower Devonian of Europe. *Zool. J. Linnean Soc.* **47**, 131-153 (1967).

136. J. A. Clack, P. E. Ahlberg, H. Blom, S. M. Finney, A new genus of Devonian tetrapod from North-East Greenland, with new information on the lower jaw of Ichthyostega. *Palaeontology* **55**, 73-86 (2012).
